# Influence of posture on prepulse inhibition and its link to postural control in healthy subjects

**DOI:** 10.1038/s41598-025-27097-4

**Published:** 2025-12-20

**Authors:** Matteo Ciocca, Sarah Hosli, Zaeem Hadi, Jingqi Hong, Yen Tai, Barry M. Seemungal

**Affiliations:** 1 Centre for Vestibular Neurology, Department of Brain Sciences, Imperial College London, London, W6 8RF UK; 2 Clinical Neuroscience Center, University Hospital Zurich, Zurich, Switzerland

**Keywords:** Prepulse inhibition, Blink reflex, Postural control, Sway, Balance control, Posturography, Neuroscience, Physiology

## Abstract

An unexpected loud sound typically elicits a reflexive eye-blink. This response is attenuated when preceded by a weaker non-startling stimulus, a phenomenon known as prepulse inhibition (PPI), which reflects sensory gating. PPI has been reported to differ between sitting and standing, suggesting a relationship with postural control. Sensory gating may dynamically suppress irrelevant inputs while preserving responsiveness to salient cues, a process likely supporting postural stability in complex environments. In this study, we investigated how PPI is modulated by postural demands and whether these changes relate to postural sway. Forty-five healthy participants underwent PPI testing while supine, standing on a hard surface, soft surface, and in tandem stance. We examined both auditory and somatosensory PPI, with and without visual input. Consistent with the hypothesis of dynamic gating, PPI was strongest during quiet standing on a firm surface, but weakened during more challenging postural conditions. While both somatosensory and auditory PPI were modulated, no significant modality-specific differences emerged. Removing visual input slightly reduced PPI but did not alter the overall pattern. These findings suggest that PPI reflects a flexible inhibitory mechanism tuned to postural context. While no causal relationship can be inferred, the results indicate a potential functional link between sensory gating and postural control, supporting future investigations into whether PPI could serve as a physiological marker of balance regulation.

**Supplementary Information:**

The online version contains supplementary material available at 10.1038/s41598-025-27097-4.

## Introduction

Standing still at a pedestrian crossing requires maintaining postural stability despite air gusts from passing lorries, loud traffic noise, and rapid visual motion from nearby vehicles. This ability relies on sensory gating, a neurophysiological mechanism that prevents sensory overload in the central nervous system by suppressing irrelevant stimuli^1^. Effective gating allows the human postural control network to prioritise proprioceptive, visual, and vestibular information necessary to optimise sway. This process involves pre-attentive neural mechanisms, primarily in the brainstem, thalamus, and cortex^2^. When sensory gating is impaired, environmental distractions may be misinterpreted as postural threats, leading to exaggerated corrective movements or even loss of balance near the kerb, with near-falls or falls posing potentially serious consequences.

Sensory gating and postural control share overlapping brainstem–cortical networks, particularly involving the pedunculopontine nucleus (PPN), a key structure in both sensory processing and gait–postural regulation^1,3^. The PPN contributes to these functions primarily by modulating peripheral sensory inputs and facilitating the adaptive adjustment of postural control in response to environmental demands. To this extent, during unchallenging postural tasks, such as standing quietly on a firm surface, it is predictable that the degree of filtering or sensory gating would be maximised. This heightened gating likely serves to suppress irrelevant environmental inputs that could otherwise interfere with the stable integration of vestibular, visual, and somatosensory information required for balance. Evidence from previous studies supports this hypothesis, showing greater sensory gating during standing compared with sitting^4^. Conversely, when postural control is challenged (such as standing on a compliant surface), it can be hypothesised that the degree of filtering is selectively reduced for posturally relevant stimuli to allow greater sensory inflow from critical modalities. At the same time, filtering for irrelevant or distracting stimuli is maintained or even enhanced to prevent sensory overload. This selective gating mechanism would enable the central nervous system to compensate for diminished reliability in one sensory modality by admitting a broader range of relevant sensory information while continuing to suppress non-essential inputs. This raises the possibility that sensory gating dynamically adapts to the demands of balance, optimising sensory information flow to maintain postural stability under varying environmental conditions.

**Fig. 1:**
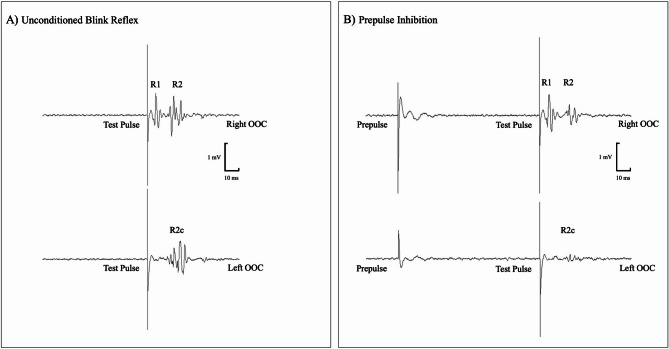
Example of an unconditioned blink reflex and somatosensory prepulse inhibition (PPI) recorded during standing. (**A**) Unrectified traces showing a typical unconditioned blink reflex response in both eyes following supraorbital nerve stimulation (test pulse). The response consists of a short-latency (9–12 ms) ipsilateral R1 component mediated by a monosynaptic pontine pathway and longer-latency (25–35 ms) bilateral R2 and R2c components mediated by polysynaptic circuits in the pons and medulla. (**B**) Unrectified traces illustrating somatosensory PPI of the blink reflex. A somatosensory electrical stimulus delivered to the ankle (prepulse) preceded the test pulse by 110 ms, resulting in marked inhibition of the R2 and R2c components, with only mild facilitation of the R1 response.

One well-established neurophysiological operational measure of sensory gating is prepulse inhibition (PPI) of the blink reflex^5^. PPI refers to the reduction in the amplitude of the blink reflex response when a non-startling stimulus (the “prepulse”) precedes a stronger, startling stimulus (the “pulse”) by a short interval^5^. PPI is typically observed as attenuation of the R2 and R2c components, although some studies also report facilitation of R1 (Fig. 1). PPN is a key structure in the control of PPI; in animals, lesions of the PPN abolish PPI^2^, whereas in humans, electrical stimulation of the PPN has been shown to evoke it^6^.

Given the shared brainstem–cortical networks underlying sensory gating and postural control^1^, and the hypothesised dynamic adaptation of gating to balance demands, we posited that PPI of the blink reflex would vary systematically with postural challenge. Specifically, we hypothesised that when postural stability is challenged, the central nervous system reduces sensory filtering to enhance the processing of relevant sensory cues required for balance maintenance.

From this hypothesis, we predicted that: (i) PPI would decrease progressively as postural task difficulty increases; (ii) somatosensory PPI (PPI_somatosensory_) would show stronger modulation with postural demand than auditory PPI (PPI_auditory_), reflecting its closer integration with sensorimotor control of balance, and (iii) task-related changes in PPI would correlate with sway parameters of static balance. To test these predictions, we systematically varied postural demand by assessing PPI across a hierarchy of tasks ranging from supine to tandem stance, thereby increasing postural challenge. We manipulated sensory feedback conditions (eyes open versus eyes closed) to examine the influence of visual input on sensory gating during balance control. Additionally, we employed different prepulse modalities (somatosensory and auditory) and interstimulus intervals to characterise modality-specific and temporal aspects of PPI modulation in relation to postural demands. This comprehensive approach enabled us to evaluate the dynamic interplay between sensory gating mechanisms and static postural control.

Our results confirmed that PPI is modulated by postural demands. We observed stronger inhibition when standing on a firm surface compared with more challenging conditions, and PPI correlated with measures of postural sway in specific conditions, potentially indicating a link between sensory gating and balance control. When sensory feedback was disrupted, a weaker inhibition was present, suggesting that the modulation of inhibitory gating is dependent on the availability of reliable sensory information for balance.

However, contrary to our initial hypothesis, we did not find statistically significant differences between PPI_somatosensory_ and PPI_auditory_ across postural conditions. This suggests that early brainstem-level sensory gating mechanisms do not selectively prioritise posturally relevant somatosensory stimuli over auditory inputs during postural challenge. Instead, inhibitory modulation appears to be adjusted globally across modalities, with modality-specific adaptations to postural demands likely occurring at higher integrative levels beyond the reflexive gating circuits. Together, these findings support the idea that sensory gating dynamically adapts to changing postural demands and sensory environments to help maintain stability, but do not provide evidence for modality-specific differences in PPI during balance control.

## Results

All participants completed the experiments without any adverse effects.

### Preliminary experiments: input-output curve for PPI_somatosensory_

To determine the most effective interstimulus interval for eliciting PPI_somatosensory_ of the blink reflex, we conducted two preliminary experiments comparing the degree of inhibition across a range of interstimulus intervals.

Data for Preliminary Experiment 1 and 2 are in Supplementary Tables 1 and 2, and Supplementary Fig. 1.

In Preliminary Experiment 1, we compared the amount of PPI_somatosensory_ for interstimulus intervals from 70 ms to 120 ms. A repeated measures one-way ANOVA showed no difference in R1 amplitude (*F*_7,120_ = 1.667, *p* = 0.124, Cohen’s *f* = 0.19). However, statistically significant differences were observed for R2 (*F*_7,120_ = 4.347, *p* = 0.0002, Cohen’s *f* = 0.427), and R2c area (*F*_7,120_ = 3.698, *p* = 0.001, Cohen’s *f* = 0.384). Post-hoc analysis revealed statistically significant differences among unconditioned and conditioned stimuli at an interstimulus interval of 110 ms (*p* = 0.0001) for both R2 and R2c area.

In Preliminary Experiment 2, we compared PPI_somatosensory_ for interstimulus intervals 110, 200, 400, 600 ms. There was no difference in R1 amplitude (*F*_4,65_ = 0.373, *p* = 0.827; Cohen’s *f* = 0). Statistically significant differences were found in R2 area (*F*_4,65_ = 5.598, *p* = 0.001; Cohen’s *f* = 0.512), with post-hoc analysis showing statistically significant differences at interstimulus intervals 110 ms (*p* = 0.003), 200 ms (*p* = 0.004), and 400 ms (*p* = 0.002). Inhibition was observed in R2c area (*F*_4,65_ = 3.316, *p* = 0.016; Cohen’s *f* = 0.363), with strongest inhibition at interstimulus intervals 110 (*p* = 0.027) and 200 ms (*p* = 0.024).

Strong inhibition at a 110 ms interstimulus interval was consistent across experiments (R2 area inhibition: 32.21% in Preliminary Experiment 1, 27.58% in Preliminary Experiment 2; R2c area inhibition: 32.55% in Preliminary Experiment 1, 28.66% in Preliminary Experiment 2). A paired t-test compared 12 subjects who participated in both experiments, showing no significant difference in PPI at 110 ms interstimulus interval between Preliminary Experiment 1 (mean 36.33%; SD = 20.10) and Preliminary Experiment 2 (mean 28.38%; SD = 15.58) [*t*_11_ = -1.457, *p* = 0.173] (See Supplementary Fig. 2). Therefore, the 110 ms interstimulus interval was selected for the main experiments with a somatosensory prepulse.

### Main experiment 1- PPI modulation under different postural conditions

In this experiment, we assessed whether PPI_somatosensory_ elicited using a 110 ms interstimulus interval was modulated by four different postural conditions (supine, standing on a hard surface with eyes open, standing on a soft surface with eyes open, and tandem stance), and whether such modulation correlated with measures of static balance.

We initially compared latencies of blink reflex responses PPI_somatosensory_ between all postural conditions. Paired t-tests comparing unconditioned and conditioned R1, R2, and R2c latencies within each postural condition revealed no differences for R1 latency (Supplementary Table 3). However, conditioned R2 and R2c latencies were consistently longer than unconditioned latencies across all postural conditions (Supplementary Table 3). Comparing latency differences between supine and standing conditions showed no significant effects of postural changes (Supplementary Table 4).

**Fig. 2:**
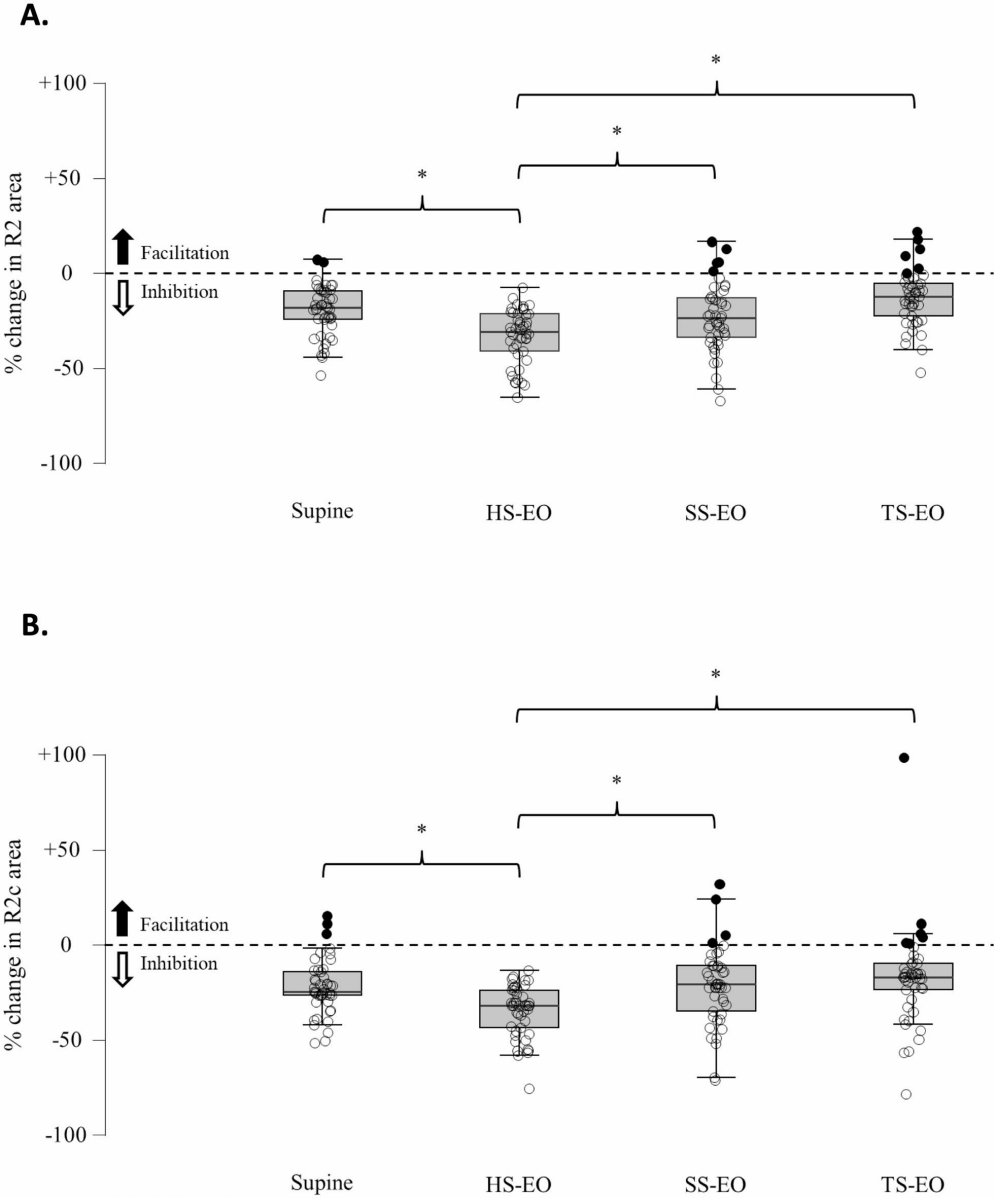
Modulation of the R2 and R2c area according to posture. Relative changes (%) in the R2 (**A**) and R2c (**B**) area are illustrated. Black filled circles represent a facilitation of the conditioned response, whereas empty filled circles represent an inhibition of the conditioned response. A statistically significant difference after Bonferroni correction is present between supine and hard surface-eyes open, hard-surface-eyes open and soft surface-eyes closed, hard surface-eyes open and tandem standing for both the R2 and R2c responses. HS-EO: hard surface – eyes open; SS-EO: soft surface – eyes open; TS-EO: tandem standing – eyes open. *: *p* < 0.0125.

We then assessed whether the amplitude and area of blink reflex responses were modulated by postural conditions (Table 1; Fig. 2).

**Table 1:** Postural modulation of the magnitude of PPI for each Blink reflex response.

		95% confidence interval mean				
	Mean percentage change	Upper	Lower	Std. error	F	*p*-value
R1 amplitude					0.068	n.s.
Supine	+ 31.74%	+ 45.75%	+ 17.72%	0.069	-	-
HS-EO	+ 32.15%	+ 48.89%	+ 15.36%	0.083	-	-
SS-EO	+ 32.71%	+ 49.69%	+ 15.73%	0.084	-	-
TS-EO	+ 28.33%	+ 41.20%	+ 15.46%	0.064	-	-
R2 area					13.589	*P* < 0.0001
Supine	-19.43%	-15.55%	-23.56%	0.019	-	-
HS-EO	-33.66%	-28.85%	-37.46%	0.021	-	-
SS-EO	-24.20%	-17.85%	-28.81%	0.027	-	-
TS-EO	-13.05%	-8.62%	-17.51%	0.022	-	-
R2c area					6.869	*p* < 0.0005
Supine	-23.43%	-17.71%	-26.42%	0.021	-	-
HS-EO	-35.23%	-30.85%	-39.02%	0.203	-	-
SS-EO	-26.87%	-15.99%	-28.19%	0.302	-	-
TS-EO	-21.54%	-10.41%	-25.41%	0.037	-	-

Mean of percentage change, confidence interval, and standard error of the amount of Inhibition for each blink reflex component is reported according to posture. Positive values reflect a facilitation, whereas negative values reflect an inhibition of the blink reflex responses. R1 amplitude shows a tendency towards facilitation similar in all the different postural conditions. Conversely, R2 and R2c area are modulated according to posture, with a stronger inhibition in the hard surface-eyes open condition and a weaker inhibition in the tandem standing condition. EO: eyes opened. EC: eyes closed. HS: hard surface; SS: soft surface; TS: tandem standing.

R1 amplitude showed no significant differences across conditions (*F*_3,176_ = 0.068, *p* = 0.977, Cohen’s *f* = 0). However, R2 and R2c area inhibition varied significantly (R2: *F*_3,176_ = 13.589, *p* < 0.0001, Cohen’s *f* = 0.458; R2c: *F*_3,176_ = 6.869, *p* < 0.0005, Cohen’s *f* = 0.313). Post-hoc analysis showed significant differences between supine and hard surface, hard surface and soft-surface, and hard-surface and tandem standing for both R2 and R2c responses (Fig. 2).

**Fig. 3:**
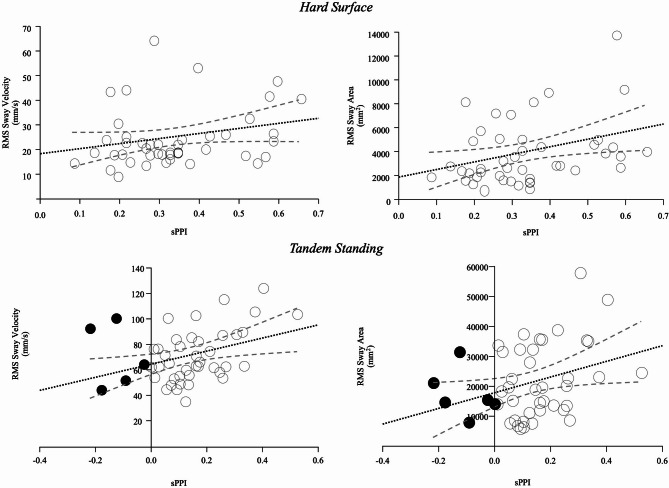
Linear correlation between PPI_somatosensory_ and sway parameters. There is a positive linear correlation between PPI_somatosensory_ and sway area while standing on hard surface and between PPI_somatosensory_ and sway velocity while tandem standing. A trend towards a positive correlation is also present between PPI_somatosensory_ and sway velocity while standing on hard surface and PPI_somatosensory_ and sway area while tandem standing. The blacked dotted line represents the regression line, while the two blue dashed lines represent the 95% confident interval.

Furthermore, to assess whether PPI_somatosensory_ modulation correlated with sway parameters, we performed a Spearman correlation analysis (Supplementary Table 5, Fig. 3), which revealed significant positive correlations between PPI_somatosensory_ and sway measures. Higher PPI_somatosensory_ levels were associated with greater sway area on hard surface and higher sway velocity while tandem standing.

Subsequently, to compare the Spearman’s correlation coefficients of sway area and velocity between hard surface, and soft surface and tandem stance, we applied Fisher’s Z-transformation to normalize the correlation values. Data are reported in Supplementary Table 5. A two-tailed t-test did not identify a significant difference between hard surface and soft surface for sway area (*p* = 0.204) and sway velocity (*p* = 0.075). Similarly, a two-tailed t-test resulted in a non-statistically significant difference between hard surface and tandem stance for both sway area (*p* = 0.868) and velocity (*p* = 0.657).

To characterise the progression of postural challenge across conditions, we analysed the delta changes in sway area and sway velocity between postural tasks (e.g., from hard surface to soft surface, and soft surface to tandem stance). As expected, both sway area and sway velocity demonstrated a progressive increase with task difficulty (see Supplementary Table 6), reflecting greater postural challenge. These incremental changes in sway confirm that the postural manipulations effectively modulated the level of challenge imposed on the postural control system.

#### Main experiment 2 – effect of visual feedback on postural PPI-modulation

In this experiment, we investigated whether visual feedback (eyes open vs. closed) modulated PPI_somatosensory_ elicited at an interstimulus interval of 110 ms, across different postural conditions (supine, hard surface, and soft surface).

We investigated the effect of visual feedback on the R1 latency and amplitude and R2/R2c latency and area across postural conditions using repeated measures ANOVA. Means and standard deviations for latency differences, R1 amplitude, and R2/R2c area inhibition are in Table 2.

**Table 2:** Visual and postural modulation of PPI for each Blink reflex response.

	Mean change (ms)	Std. deviation		Percentage change (%)	Std. Deviation
R1 latency			R1 amplitude		
Supine-EO	+ 0.0256	0.3049	Supine-EO	+ 36.18%	32.507
Supine-EC	+ 0.1250	0.5063	Supine-EC	+ 21.13%	40.655
HS-EO	+ 0.1122	0.3775	HS-EO	+ 35.11%	40.074
HS-EC	+ 0.1967	0.3856	HS-EC	+ 15.92%	25.011
SS-EO	+ 0.1106	0.5470	SS-EO	+ 42.68%	66.266
SS-EC	+ 0.1783	0.4083	SS-EC	+ 13.29%	25.195
R2 latency			R2 area		
Supine-EO	+ 1.253	1.3664	Supine-EO	-16.07%	9.77
Supine-EC	+ 1.335	2.0743	Supine-EC	-19.80%	16.33
HS-EO	+ 1.286	1.6822	HS-EO	-29.50%	14.91
HS-EC	+ 0.762	1.5538	HS-EC	-25.52%	18.70
SS-EO	+ 1.318	2.3707	SS-EO	-18.52%	14.41
SS-EC	+ 1.572	1.3885	SS-EC	-16.18%	23.05
R2c latency			R2c area		
Supine-EO	+ 1.5211	1.5310	Supine-EO	-19.42%	7.47
Supine-EC	+ 0.6856	1.6388	Supine-EC	-21.62%	20.10
HS-EO	+ 1.5950	1.5267	HS-EO	-29.33%	10.21
HS-EC	+ 1.1506	1.7748	HS-EC	-30.74%	18.49
SS-EO	+ 1.3817	3.8917	SS-EO	-16.47%	9.08
SS-EC	+ 2.257	1.6237	SS-EC	-17.17%	26.70

Mean changes and standard deviation in latency (ms) for R1, R2, and R2c are reported according to the presence (Eye opened) or absence (eyes closed) of Visual feedback and according to the postural condition. Relative changes (%) and standard deviation for the R1 amplitude (mV) and R2 and R2c area (mV*ms) are reported. EO: eyes opened. EC: eyes closed. HS: hard surface; SS: soft surface.

Visual feedback and postural conditions had no significant effect on R1, R2, or R2c latency (Supplementary Table 7).

R1 amplitude was significantly larger with eyes open than eyes closed (*p* = 0.029; Table 2, Supplementary Table 7, Supplementary Fig. 3). Neither postural conditions nor their interaction with visual feedback significantly influenced R1 amplitude.

The effect of visual feedback on R2 and R2c areas was not significant. However, postural condition alone significantly influenced R2 and R2c inhibition. Post-hoc pairwise comparisons showed stronger inhibition on a hard surface compared to supine (R2: *p* = 0.005; R2c: *p* = 0.007) and soft surfaces (R2: *p* = 0.001; R2c: *p* = 0.001). Separate one-way ANOVA analyses confirmed significant differences in PPI_somatosensory_ between supine and hard-surface standing with eyes open (*F*_2,54_ = 4.389, *p* = 0.016, Cohen’s *f* = 0.345; R2: *p* = 0.02; R2c: *p* = 0.005). No significant differences were found for eyes-closed conditions (*F*_2,57_ = 1.070, *p* = 0.350, Cohen’s *f* = 0.05) (Table 2, Supplementary Table 7, Supplementary Fig. 3).

Pearson correlations between PPI_somatosensory_ and sway velocity/area were not significant (Supplementary Table 8).

### Main experiment 3 – effect of different interstimulus intervals on postural PPI-modulation

This experiment assessed whether the modulation of PPI_somatosensory_ by postural condition varied according to the interstimulus interval between the prepulse and test pulse. Two comparisons were made: a shorter interstimulus interval (80 ms) versus the optimal interstimulus interval (110 ms) in Experiment 3a, and a longer interstimulus interval (200 ms) versus the optimal interstimulus interval (110 ms) in Experiment 3b.

**Fig. 4:**
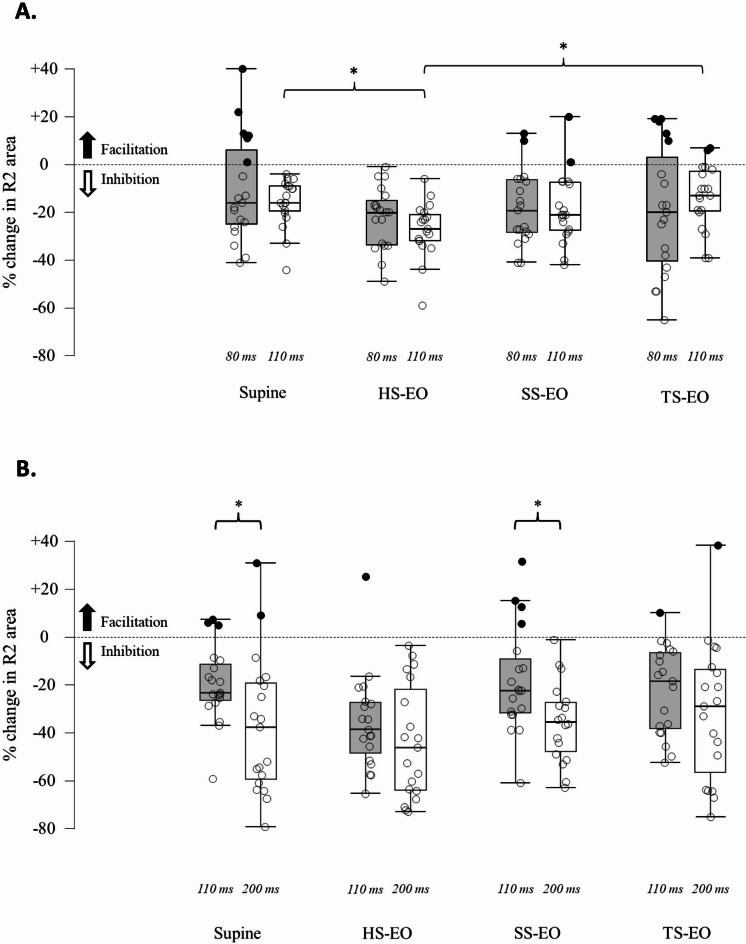
Modulation of the R2 area according to posture and two different interstimulus intervals. Relative changes (%) in the R2 area (mV*ms) are illustrated. Black filled circles represent a facilitation of the conditioned response, whereas empty circles represent an inhibition of the conditioned response. In A, a statistically significant difference after Bonferroni correction is present between supine and hard surface and hard-surface and tandem standing at 110 ms interstimulus interval, whereas a trend towards an inhibition is present at an interstimulus interval of 80 ms. In B, a statistically significant difference is present according to the interstimulus interval deployed in the supine and soft surface-eyes open conditions. Inhibition is present with both interstimulus intervals, with a stronger inhibition for the 200 ms interstimulus interval, but a lack of postural modulation. HS-EO: hard surface – eyes open; SS-EO: soft surface – eyes open; TS-EO: tandem standing – eyes open. *: *p* < 0.05.

For Experiments 3a and 3b, repeated measures ANOVA results and descriptive statistics are in Table 3; Fig. 4, and Supplementary Tables 9 and 10.

**Table 3:** Postural modulation of ppisomatosensory according to the interstimulus interval deployed for each Blink reflex response.

		Percentage change (%)	Std. deviation			Percentage change (%)	Std. deviation
R1 amplitude				R1 amplitude			
Supine	80 ms	+ 41.72%	33.67	Supine	110 ms	+ 39.75%	63.22
	110 ms	+ 36.18%	33.39		200 ms	+ 31.86%	56.18
HS	80 ms	+ 19.61%	33.97	HS	110 ms	+ 32.25%	73.10
	110 ms	+ 35.11%	41.17		200 ms	+ 30.41%	42.74
SS	80 ms	+ 38.59%	65.67	SS	110 ms	+ 18.15%	38.52
	110 ms	+ 42.68%	68.08		200 ms	+ 36.74%	57.91
TS	80 ms	+ 13.69%	40.34	TS	110 ms	+ 36.06%	28.97
	110 ms	+ 30.59%	49.83		200 ms	+ 47.50%	57.59
R2 area				R2 area			
Supine	80 ms	-10.54%	21.26	Supine	110 ms	-19.71%	16.00
	110 ms	-16.10%	9.7		200 ms	-37.06%	28.67
HS	80 ms	-22.13%	12.63	HS	110 ms	-35.79%	20.15
	110 ms	-27.27%	11.16		200 ms	-43.37%	23.86
SS	80 ms	-18.12%	14.98	SS	110 ms	-18.01%	22.11
	110 ms	-18.52%	14.41		200 ms	-37.61%	19.32
TS	80 ms	-19.35%	26.11	TS	110 ms	-22.31%	18.60
	110 ms	-13.58%	12.93		200 ms	-31.16%	28.94
R2c amplitude				R2c area			
Supine	80 ms	-6.39%	25.04	Supine	110 ms	-26.59%	26.62
	110 ms	-19.42%	7.47		200 ms	-41.06%	33.11
HS	80 ms	-18.96%	19.30	HS	110 ms	-27.75%	25.66
	110 ms	-29.33%	10.21		200 ms	-39.59%	24.76
SS	80 ms	-19.17%	21.87	SS	110 ms	-23.89%	26.66
	110 ms	-16.47%	9.08		200 ms	-39.53%	24.45
TS	80 ms	-17.19%	34.55	TS	110 ms	-30.63%	23.80
	110 ms	-13.19%	12.05		200 ms	-39.12%	23.57

Percentage changes (%) and standard deviation for R1, R2, and R2c are reported according to the specified interstimulus interval (80 ms vs. 110 ms and 110 ms vs. 200 ms) and according to the postural condition. HS: hard surface; SS: soft surface, TS: tandem standing.

#### Experiment 3a – comparing 80 ms vs. 110 ms

A significant latency difference was found between interstimulus intervals 80 ms and 110 ms (*F*_1,19_ = 8.092; *p* = 0.010; Cohen’s *f* = 0.581). T-tests showed longer latency for 110 ms interstimulus interval in R2 (*t* = -2.149; *p* = 0.045) and R2c (*t* = -2.425; *p* = 0.025). Statistical analysis showed no significant differences in latencies across postural conditions for either interstimulus interval.

R1 amplitude modulation across postural conditions was not significant (*F*_1,18_ = 1.143; *p* = 0.340; Cohen’s *f* = 0.08). The interstimulus interval did not significantly affect R2 (*F*_1,18_ = 1.582; *p* = 0.204; Cohen’s *f* = 0.17) or R2c area (*F*_1,18_ = 2.407; *p* = 0.077; Cohen’s *f* = 0.265).

Separate one-way ANOVA confirmed stronger PPI_somatosensory_ at a 110 ms interstimulus interval for both R2 (*F*_3,72_ = 4.308; *p* = 0.007; Cohen’s *f* = 0.361) and R2c (*F*_3,72_ = 9.129; *p* < 0.0001; Cohen’s *f* = 0.566). Post-hoc tests revealed stronger inhibition on a hard surface compared to supine (R2: *p* = 0.044; R2c: *p* = 0.019) and tandem standing (R2: *p* = 0.007; R2c: *p* < 0.0001). No significant differences were found for R2 (*F*_3,72_ = 1.142; *p* = 0.338; Cohen’s *f* = 0.07) or R2c (*F*_3,72_ = 0.993; *p* = 0.401; Cohen’s *f* = 0) at an 80 ms interstimulus interval.

#### Experiment 3b – comparing 110 ms vs. 200 ms

No significant latency differences were found between 110 ms and 200 ms interstimulus intervals for R1 (*F*_3,54_ = 0.580; *p* = 0.631; Cohen’s *f* = 0), R2 (*F*_3,54_ = 1.878; *p* = 0.144; Cohen’s *f* = 0.213), or R2c (*F*_3,54_ = 2.509; *p* = 0.068; Cohen’s *f* = 0.122).

The interaction between interstimulus interval and postural condition was not significant for R1 amplitude (*F*_3,54_ = 1.307; *p* = 0.282; Cohen’s *f* = 0.126), R2 (*F*_3,54_ = 2.458; *p* = 0.073; Cohen’s *f* = 0.275), or R2c areas (*F* = 0.813; *p* = 0.492; Cohen’s *f* = 0). However, paired sample t-tests showed stronger inhibition with a 200 ms interstimulus interval in all postural conditions (Supplementary Table 10, Fig. 4).

Main experiment 4 – effect of prepulse modality on postural PPI-modulationThis experiment investigated PPI_auditory_ at an interstimulus interval of 100 ms across the same postural conditions tested in Experiments 1 and 2. Therefore, participants were assessed in four postural positions: supine, standing on a hard surface, standing on a soft surface, and tandem stance on a hard surface. Each posture was tested under both eyes open and eyes closed conditions, allowing evaluation of the combined effects of posture and visual feedback on PPI_auditory_.

We compared the latency of unconditioned and conditioned R1, R2, and R2c responses within each postural condition using paired t-tests. No significant difference was found for R1 latency. However, conditioned R2 and R2c latencies were consistently longer than unconditioned latencies across all postural conditions (Supplementary Table 11). Postural changes did not significantly affect these latency differences (Supplementary Table 12).

**Fig. 5:**
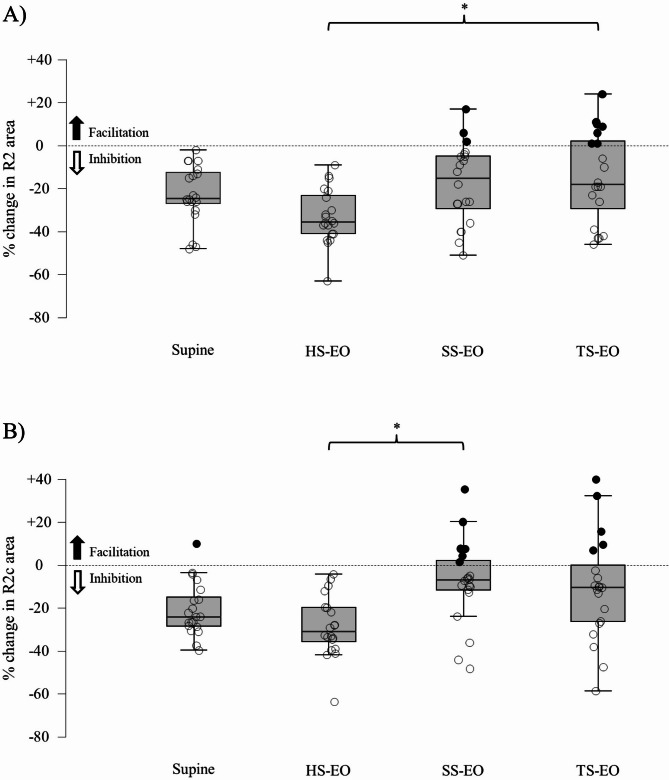
Modulation of PPI_auditory_ according to posture. Relative changes (%) in the R2 (**A**) and R2c (**B**) area are illustrated. Black filled circles represent a facilitation of the conditioned response, whereas empty circles represent an inhibition of the conditioned response. A statistically significant difference after Bonferroni correction is present between hard surface-eyes open and tandem stance for the R2 response and between hard surface-eyes open and soft-surface-eyes open for the R2c response. HS: hard surface; SS: soft surface; TS: tandem standing. *: *p* < 0.0125.

We then examined the modulation of blink reflex responses across postural conditions, focusing on R1 amplitude and R2 and R2c area. One-way ANOVA showed no significant difference in R1 amplitude across postural conditions (*F*_3,79_ = 0.929; *p* = 0.431; Cohen’s *f* = 0). Conversely, prepulse inhibition of R2 and R2c area varied significantly (R2: *F*_3,79_ = 4.476; *p* = 0.006; Cohen’s *f* = 0.361; R2c: *F*_3,79_ = 5.266; *p* = 0.002; Cohen’s *f* = 0.399). Post-hoc tests revealed significant differences for R2 between hard surface-eyes open and tandem stance (*p* = 0.006) and for R2c between hard surface-eyes open and soft surface-eyes open (*p* = 0.004) (Supplementary Tables 13 and Fig. 5).

A one-way ANOVA did not show any significant difference in the amplitude of R1 across different postural conditions (*F*_3,79_ = 0.929, *p* = 0.431, Cohen’s *f* = 0). Conversely, prepulse inhibition of the R2 and R2c area was modulated across different postural conditions (R2: *F*_3,79_ = 4.476, *p* = 0.006, Cohen’s *f* = 0.361; R2c: *F*_3,79_ = 5.266, *p* = 0.002, Cohen’s *f* = 0. 399). A post-hoc analysis revealed a statistically significant difference between hard surface-eyes open and tandem stance for the R2 area (*p* = 0.006), and between hard surface-eyes open and soft surface-eyes open for the R2c area (*p* = 0.004) (see Fig. 5).

Subsequently, we evaluated the effect of visual feedback (eyes open vs. eyes closed) on PPI_auditory_ using repeated measures ANOVA. No significant differences were found in R1 amplitude (*F*_2,38_ = 0.554; *p* = 0.579), R2 area (*F*_3,38_ = 0.793; *p* = 0.460), or R2c area (*F*_2,38_ = 1.715; *p* = 0.194).

Finally, a Spearman correlation analysis revealed a significant positive correlation between PPI_auditory_ and sway area while tandem standing, with higher PPI_auditory_ associated with greater sway area (Supplementary Fig. 4; Supplementary Table 14).

### Cross modal analysis of PPI

To determine whether inhibitory sensory gating differs between sensory modalities, we compared PPI_auditory_ and PPI_somatosensory_ PPI values in participants who underwent both modalities across all postural conditions. This analysis was performed to test our hypothesis that somatosensory prepulses, being more directly related to postural feedback, would exhibit stronger modulation under challenging balance conditions compared with auditory prepulses.

We examined the modulation of blink reflex responses across postural conditions, focusing on R1 amplitude and R2 and R2c area, and comparing PPI_somatosensory_ to PPI_auditory_ (Supplementary Table 15). Contrary to our hypothesis, the analysis revealed no statistically significant difference between PPI_somatosensory_ and PPI_auditory_ across postural conditions (R1: *F*_3,54_ = 1.198; *p* = 0.319; R2: *F*_3,54_ = 0.991; *p* = 0.404; R2c: *F*_3,54_ = 0.664; *p* = 0.578).

## Discussion

In this study, we investigated the link between PPI and postural control. Our findings revealed that (a) PPI is modulated by postural conditions, with stronger inhibition when standing on hard surface. Notably, both PPI_somatosensory_ and PPI_auditory_ exhibit this modulation, and contrary to our initial hypothesis, no statistically significant difference was observed between the two modalities across postural conditions. This suggests that PPI modulation is centrally regulated and operates similarly across sensory modalities in this postural paradigm, without preferential enhancement for somatosensory inputs. (b) PPI shows condition-specific correlations with sway parameters. Specifically, on hard surface and while tandem standing, PPI_somatosensory_ positively correlated with sway area and/or velocity in some conditions, suggesting that, under particular postural demands, greater sway may be associated with stronger inhibition. A weaker and less consistent association was observed between PPI_auditory_ and sway during tandem stance. These findings indicate a context-dependent interaction between sensorimotor gating and postural sway rather than a uniform relationship across tasks or modalities. The positive correlation between PPI and sway on firm surface and in tandem stance should not necessarily be interpreted as evidence that increased instability enhances inhibition. Instead, it may reflect individual differences in the efficiency of sensorimotor gating, with higher PPI representing a compensatory mechanism that supports postural robustness under varying balance demands. Nevertheless, these associations were modest overall and should be interpreted with caution.

(c) Peripheral sensory feedback perturbations reduce inhibition. Standing on soft surface or with eyes closed reduces inhibition, with a greater reduction on soft surface, highlighting the role of sensory feedback in postural variations of PPI. (d) PPI_somatosensory_ modulation is time-specific, peaking at 110 ms and disappearing by 200 ms, indicating time-dependent sensitivity in PPI changes.

The observed difference in PPI across different postural conditions highlights a potential functional relationship between sensorimotor gating and postural control strategies, although causality cannot be inferred from the present cross-sectional design.

The magnitude of inhibition varies across postural conditions, as previously observed^4^. Our study confirms this and further demonstrates that PPI modulation reflects the adaptability of sensorimotor integration within the postural control network. PPI likely prevents sensory overflow during less challenging tasks (e.g., standing on hard surface) and enhances peripheral feedback during more demanding tasks (e.g., standing on soft surface or tandem standing). Neural activity within shared structures, such as the PPN and the caudal pontine reticular nucleus, is critical in this process, with concurrent bottom-up and top-down control mechanisms.

PPI varies with sensory feedback perturbations, such as those affecting proprioceptive (e.g. soft surface and tandem standing), or visual (e.g. eyes closed) systems. In these conditions, reduced inhibition likely reflects decreased sensory filtering to maintain balance when peripheral inputs are disrupted^7,8^. This result is in line with previous data showing how the startle reflex is reduced when the afferent volley comes from the lower limbs, likely a “defensive” mechanism of the postural control network to guarantee balance control in challenging situations^4,8^.

However, interpretation of PPI changes in these postural conditions requires caution. For example, standing on soft surface or in tandem stance does not impair proprioceptive afferents per se^9,10^. Rather, it alters the mechanical interaction between the foot and support surface, introducing a mismatch between expected and actual force-feedback dynamics^11,12^. As such, the reduced amount of inhibition observed on foam and in tandem stance may not solely reflect postural challenge, but instead a shift in sensorimotor strategy due to unfamiliar task mechanics^13,14^, akin to adaptation processes observed in novel force-field paradigms^15^. This interpretation is compatible with a motor learning framework, in which attentional demand and sensory reweighting play a significant role^16,17^. Accordingly, reduced inhibition during challenging tasks might arise from heightened attentional engagement or adaptive recalibration, rather than a linear relationship with postural instability.

The correlation between PPI and sway parameters further suggests that PPI reflects activity within the postural control network. Notably, PPI correlates with sway when proprioceptive feedback remains intact but not when it is perturbed. This indicates that PPI primarily reflects proprioceptive feedback integration, highlighting the importance of proprioceptive inputs from muscles, joints, and cutaneous receptors^7^. The time-dependent nature of PPI modulation, observed at specific interstimulus intervals (80–110 ms), supports this interpretation, as it aligns with the temporal integration of proprioceptive feedback at cortical and subcortical levels^4^. However, the absence of PPI modulation at longer interstimulus intervals likely reflects central top-down mechanisms. This might indicate the involvement of structures modulating PPI, such as limbic structures^18^, which are less involved in regulating balance during quiet standing in young healthy subjects^19^.

Previously, we hypothesised that somatosensory prepulses would exhibit stronger modulation under postural challenge compared with auditory prepulses. Contrary to this, our findings showed no statistically significant difference between the modalities, indicating that early brainstem-level gating mechanisms respond similarly to somatosensory and auditory stimuli during static balance tasks. This challenges the notion that the relevance of the prepulse stimulus (posturally related vs. non-related) influences inhibitory control, suggesting instead a centrally tuned, modality-independent gating process.

In summary, our results seem to support the coexistence of a central tuning hypothesis and a peripheral feedback hypothesis, which are mutually contributing to modulate PPI when balancing.

The possible involvement of structures modulating PPI offers an alternative interpretation of our findings, particularly regarding task difficulty. On hard surface, participants with greater inhibition exhibited more sway. However, this task did not challenge the postural control network, as none of the participants reported feelings of instability or unsteadiness, although we did not specifically query this. We therefore infer that all participants maintained optimal postural control, with PPI potentially serving as a surrogate marker of a form of “balance reserve”, where individuals with stronger inhibition might better adapt and reduce sway during more demanding tasks. This would align with the concept of “cognitive reserve” as representing the physiological robustness within the (postural control) brain network^20,21^. Nevertheless, we observed no correlation between changes in PPI from hard surface to tandem stance and the corresponding changes in sway area and velocity. Consequently, this hypothesis cannot be fully supported.

A limitation in this context is the lack of control for individual perception of task difficulty. While sway area and velocity exhibited a progressive increase from standing on a hard surface to a soft surface, and subsequently to tandem stance, likely indicative of escalating postural challenge, participants were not formally queried regarding their perceived instability or the effort required to maintain postural control. Instead, we assumed that the transition from hard surface to tandem stance posed an increased challenge for all participants, which may not have been uniformly true. Additionally, subjective perception of stability does not invariably align with objective balance measures; increased arousal and primed expectations of difficulty can lead to a dissociation between self-reported instability and sway parameters^22,23^. To fully address this question, both subjective ratings and objective measures would have been required. The absence of such subjective measures in the present study consequently limits our capacity to examine this relationship directly.

Conversely, changes in correlation between PPI_somatosensory_ and sway parameters may reflect a maladaptive response, where cognitively-driven mechanisms assume control over posture from automatic processes^24^. In this scenario, PPI may only reliably reflect postural control when subcortical automatic processes dominate. The reduction in correlation when transitioning from hard surface to soft surface supports this hypothesis. An important limitation to this interpretation is that we did not test PPI while participants were standing and concurrently performing a cognitive task. A dual-task paradigm would have allowed for a clearer understanding of whether cognitive control interferes with automatic postural processes and whether this results in a maladaptive response. The absence of such a challenge to cognitive processes in this study limits the ability to definitively assess whether higher cognitive demands affect PPI’s role in postural control. Further studies incorporating a dual-task approach and larger sample sizes are needed to clarify this relationship.

PPI modulation may partly reflect attentional influences on the postural control network. The PPN and the caudal pontine reticular nucleus, critical in both networks, are primarily involved in automatic balance control. However, under challenging conditions, attentional resources are increasingly recruited to integrate visual, vestibular, and proprioceptive inputs needed to maintain stability^25,26^. When sensory inputs are limited, such as on soft surface or with eyes closed, attentional demand increases, potentially diverting resources from automatic postural mechanisms and leading to compromised stability^27,28^. Excessive attentional focus on stabilising posture can disrupt compensatory neural responses, leading to increased instability^29^. This possibility is consistent with our findings of reduced PPI when sensory feedback (e.g., soft surface), visual input (e.g., eyes closed), or vestibular feedback (e.g., tandem stance) are disrupted, suggesting that attentional resources are redirected to managing these sensory challenges.

PPI is sensitive to attentional modulation^30,31^ with increased attention to the prepulse enhancing inhibition and attentional diversion diminishing it. However, some studies report no PPI modulation under low-attention tasks^32^. Thus, under certain conditions, changes in PPI during different postural tasks could reflect attention-related modulation. To this extent, our findings of reduced PPI during sensory feedback alteration (e.g., soft surface, eyes closed) are consistent with a shift of attentional resources toward balance maintenance.

Importantly, when the postural task becomes extremely challenging, the balance control mechanism shifts from an automatic to cognitively driven processes, as observed in older adults with an age-related decline in balance and somatosensory function^24,33^. This transition may affect the PPI network similarly: reduced ability to dynamically adjust sensory filtering could contribute to sensory overflow and diminished postural stability, thereby increasing fall risk.

## Conclusion

PPI likely reflects sensory gating processes that are engaged during postural control and influence the integration of peripheral sensory feedback. It is conceivable that PPI might be used as a surrogate marker of activity within the postural control network, although further studies will need to confirm this possibility.

Modulation of peripheral sensory feedback, particularly proprioceptive input, significantly influences PPI, underscoring its role as a marker of sensorimotor integration during standing. However, central modulation also plays a role, as evidenced by PPI’s independence from the prepulse type. This suggests that the PPI network’s response to postural changes is inherently structured within the network itself. Nevertheless, the interpretation of PPI modulation as a marker of specific neural circuits involved in postural control must remain cautious in the absence of direct neurophysiological or imaging data. Behavioural correlations alone cannot support strong inferences about brain connectivity or regional activation. Future studies using functional neuroimaging or electrophysiological techniques will be essential to validate these mechanistic models and elucidate the neural substrates underlying PPI modulation during postural control. Additionally, modulation of PPI under conditions such as standing on foam or during tandem stance may be partly driven by novelty, sensorimotor adaptation, and increased attentional demand, rather than by postural instability per se. These alternative interpretations should be systematically addressed in future research.

## Methods

The study was conducted according to the guidelines of the Declaration of Helsinki, and approved by the Ethics Committee of London Harrow (REC reference 21/LO/0112, date of approval 21/04/2021). Informed consent was obtained from all subjects involved in the study. Adverse events were monitored throughout all experiments by actively querying participants immediately after each testing session, supplemented by self-reporting of any discomfort or unexpected symptoms.

### Blink reflex

The electrically-elicited blink reflex was used in this study. Surface electrodes were used to deliver supraorbital nerve stimuli, with the cathode placed over the supraorbital notch and the anode positioned 3 cm away along the course of the nerve on the ipsilateral forehead. Percutaneous stimuli lasting 0.2 ms were applied to the right supraorbital nerve at irregular intervals of 20 s. The stimulus intensity was set at three times the R2 motor threshold, defined as the minimum stimulus intensity able to elicit a 50 uV baseline-to-peak amplitude in at least 4 out of 8 consecutive electromyographic (EMG) responses.

The blink reflex response was recorded via EMG of the orbicularis oculi muscles. The active recording electrodes were positioned bilaterally on the lower eyelid, halfway between the inner and outer edges of the orbit, while the corresponding reference electrode was placed on the ipsilateral temple on each side. Amplification of the responses (x1000) was performed using a Digitimer D360R-4 device (Digitimer, Welwyn Garden City, UK). The signal was filtered using a frequency range of 30 Hz to 3 kHz^34^. The sampling rate was set at 1000 Hz for each EMG channel. Data acquisition was carried out using Signal software, version 7.02 (Cambridge Electronic Devices, Cambridge, UK), and recorded on a laptop computer. For R1 analysis, the amplitude of the EMG response was measured on the unrectified trace, with cursors manually positioned to capture the highest and lowest peaks of the R1 component. For R2 and R2c analysis, EMG signals were rectified, and response onset and offset were identified by visual inspection of the rectified traces, following established criteria^34–36^. The onset of R2 was defined as the first point where EMG activity rose consistently above baseline within the expected latency window, and the offset as the point where the signal returned to baseline^34–36^. Traces contaminated by EMG artifacts were excluded from analysis. Artifacts were defined as EMG activity exceeding 400 µV (peak-to-peak) and sustained for > 100 ms within 200 ms before or after stimulus onset, with morphology inconsistent with typical R1 or R2 responses. All trials were visually inspected for artifacts immediately after acquisition, and contaminated traces were discarded prior to analysis. If two or more traces within a single acquisition were affected, the entire acquisition was discarded and reacquired immediately.

### Prepulse inhibition

Somatosensory stimuli: Conditioning somatosensory stimuli (prepulses) consisted of single pulses of electrical stimulation lasting 0.2 ms, delivered via bipolar electrodes to the sural nerve at the ankle. Specifically, the electrode placement was posterior to the lateral malleolus, secured in position using a Velcro strap. The intensity was set at twice the sensory threshold, defined as the minimum stimulus intensity perceived in at least 4 out of 8 consecutive stimuli, determined through both ascending and descending stepwise approaches. This intensity level was confirmed before each acquisition for every postural condition.

Auditory stimuli: The auditory conditioning stimuli comprised a 500 Hz tone, lasting 20 microseconds, and with an intensity of 70 dB. These stimuli were administered bilaterally using a pair of headphones. Throughout the tasks, a background room noise of around 35–40 dB was consistently present. The tones were produced via a custom-made tone generator.

### Posturography

Postural sway was recorded using a force platform (51.5 × 51.5 × 18 cm), containing four force sensors (Hottinger Baldwin Messtechnik – type U1Y – 100 kg – 2 mV/V), in a rectangular configuration (250 Hz sampling rate). Each acquisition was performed while recording the blink reflex, lasting a total of 200 s. Each sway trace was screened for large deviations indicative of weight shifts or transient loss of balance requiring corrective movements. No traces were discarded, as no such deviations were observed during any acquisition.

Centre of pressure trajectories were analysed separately in the mediolateral and anteroposterior planes, and from these we derived two standard static sway metrics: (i) sway area, defined as the 95% confidence ellipse area enclosing the CoP trajectory, and (ii) sway velocity, calculated as the mean CoP path length divided by trial duration. These metrics reflect the overall magnitude and temporal dynamics of involuntary body-centre oscillations during quiet stance.

Participants were assessed under different postural conditions of increasing difficulty: (i) eyes open on a hard surface, which served as a baseline condition with full sensory input; (ii) eyes closed on a hard surface, in which visual input was removed, increasing reliance on somatosensory and vestibular feedback; (iii) eyes open on a soft surface, which reduces somatosensory precision from the plantar surface and challenges proprioceptive integration; and (iv) eyes closed on soft surface, combining visual deprivation with somatosensory attenuation, thereby maximally engaging vestibular contributions. Tandem stance, in which participants stand heel to toe with feet aligned along a narrow line, markedly reduces the base of support and increases postural control demands. In tandem stance, although somatosensory feedback remains intact, sensory processing is re-weighted to maintain balance with limited stability margins.

### Experimental design

#### Preliminary experiments

As data on the best interstimulus interval to evoke a consistent PPI_somatosensory_ from the leg are scant in the literature, we initially set up an input-output curve exploring interstimulus intervals ranging from 70 to 120 ms in steps of 10 ms; in addition, we tested an interstimulus interval of 116 ms, as this was previously used by Versace et al.^4^. Therefore, a total of 7 conditions were tested in 16 young healthy subjects (10 females; aged 21–33) (Preliminary Experiment 1). Additionally, we expanded the investigations in the input-out curve for PPI_somatosensory_ by investigating three longer interstimulus intervals, namely 200, 400 and 600 ms, and we ran a test-retest reliability protocol by retesting the interstimulus interval that in the first preliminary experiment showed the strongest inhibition. Therefore, a total of four conditions were tested in 14 young healthy subjects (8 females; aged 21–33) (Preliminary Experiment 2).

#### Main experiments

##### Main experiment 1 – PPI modulation under different postural conditions

A total of 45 subjects (16 females; aged 21–40) participated in this experiment. The interstimulus interval indicating the strongest inhibition from preliminary experiments 1 and 2 (i.e. 110 ms) was used in this experiment.

Subjects were tested in 4 different conditions: (1) supine – eyes open; (2) standing on hard surface – eyes open; (3) standing on soft surface – eyes open; and (4) standing on hard surface in tandem stance. The order of testing was pseudorandom, as the supine condition was always tested first, followed by a standing condition randomly assigned using a randomisation list. For each postural condition, 5 unconditioned and 5 conditioned blink reflexes were acquired in a random order (total of 10 responses). Participants stood on a soft rectangular foam pad (50 × 41 × 6 cm, Airex) for the soft condition. Participants were allowed to rest between conditions to minimise fatigue.

In this experiment, postural control varies according to the different peripheral feedback in each condition. In the hard surface–eyes open condition, visual, proprioceptive, and vestibular input contributes to postural control. In the soft surface–eyes open condition, primarily visual and vestibular input contributes to postural control while the proprioceptive feedback is reduced. By using the tandem condition, we chose to minimise the antero-posterior oscillations, highlighting the medio-lateral sway^37^. Visual input mainly contributes to postural control when tandem standing, while both proprioceptive and vestibular input are perturbed.

While acquiring PPI_somatosensory_ in different postural conditions, we concomitantly measured static balance using a force platform. All subjects were asked to stand on a force platform with their arms hanging loosely by their sides, with heels 8 cm apart for the hard and soft-surface balance measure, and in the heel-to-toe position for the tandem standing. Area and velocity measures of sway were calculated for each condition, and were correlated with the amount of PPI for each subject.

##### Main experiment 2 – effect of visual feedback on postural PPI-modulation

A total of 18 subjects (10 females; aged 21–37) participated in this experiment. Subjects were tested in a total of 6 different conditions: (1) supine – eyes open; (2) supine – eyes closed; (3) hard surface-eyes open; (4) hard surface – eyes closed; (5) soft surface-eyes open; and (6) soft surface – eyes closed. The order of testing was pseudorandom, as the supine condition was always tested first, followed by a standing condition randomly assigned using a randomisation list. For each condition, 5 unconditioned and 5 conditioned blink reflexes were acquired in a random order (total of 10 responses). Participants stood on a soft rectangular foam pad for the soft condition. Participants were allowed to rest between conditions to minimise fatigue. Once again, we concomitantly measured static balance for each condition by means of postural sway using a force platform.

##### Main experiment 3 – effect of different interstimulus intervals on postural PPI-modulation

In this experiment, we investigated whether PPI_somatosensory_ modulation according to posture depends on the timing between the prepulse and the test pulse. We divided this experiment in two parts, namely 3a and 3b. In experiment 3a, we compared the optimal interstimulus interval (i.e. 110ms) against a shorter, suboptimal interstimulus interval of 80 ms. In experiment 3b, we compared the optimal interstimulus interval with a longer interval of 200 ms. A total of 19 subjects (10 females; aged 21–37) participated in experiment 3a, while a total of 20 subjects (all males; aged 21–32) participated in experiment 3b.

##### Experiment 4 – effect of prepulse modality on postural PPI-modulation

A total of 20 subjects (10 females, aged 21–30) participated in this experiment. We run the same tests deployed in experiments 1 and 2 using an auditory prepulse and an interstimulus interval of 100 ms.

### Data analysis and statistics

In all experiments, we measured latency of the R1, R2, and R2c responses, amplitude of R1, and area-under-the-curve (area) of the R2 and R2c components of the blink reflex in each single rectified trace in control and test trials. We then normalised the amount of inhibition for each subject, and we averaged the result per subject and per condition. We reported the percentage of facilitation/inhibition for the R1 and R2/R2c according to these formulas: for R1$\:\left(\frac{\text{R}1\text{c}\text{o}\text{n}\text{d}\text{i}\text{t}\text{i}\text{o}\text{n}\text{e}\text{d}\:\text{a}\text{m}\text{p}\text{l}\text{i}\text{t}\text{u}\text{d}\text{e}}{\text{R}1\text{u}\text{n}\text{c}\text{o}\text{n}\text{d}\text{i}\text{t}\text{i}\text{o}\text{n}\text{e}\text{d}\:\text{a}\text{m}\text{p}\text{l}\text{i}\text{t}\text{u}\text{d}\text{e}}-1\right)*100$, for R2/R2c ($\:\frac{\text{R}2\text{c}\text{o}\text{n}\text{d}\text{i}\text{t}\text{i}\text{o}\text{n}\text{e}\text{d}\:\text{a}\text{r}\text{e}\text{a}}{\text{R}2\text{u}\text{n}\text{c}\text{o}\text{n}\text{d}\text{i}\text{t}\text{i}\text{o}\text{n}\text{e}\text{d}\:\text{a}\text{r}\text{e}\text{a}}-1)*100$. A positive value reflects a facilitation, a negative value an inhibition.

For experiments 1, a one-way fixed-effects ANOVA was used to assess differences in PPI_somatosensory_ across postural conditions. An a-priori power analysis was conducted using G*Power version 3.1.9.6^38^ to determine the minimum required sample size. Based on the effect sizes reported in previous literature^4^, we assumed a moderately large effect size (Cohen’s *f* = 0.5), consistent with prior work in healthy participants using analogous paradigms. Under this assumption, to achieve 80% power at α = 0.05 for a fixed-effects one-way analysis of variance (ANOVA), a minimum sample size of *N* = 48 was required. Thus, the obtained sample size of *N* = 45 for experiment 1 was deemed sufficient given the within-subject design and the conservative effect size estimate.

For Experiment 2, which compared the modulation of PPI_somatosensory_ across four postural conditions (supine, hard, soft, tandem) and two visual conditions (eyes open, eyes closed), an a priori power analysis was conducted for a within-subjects repeated measures ANOVA (within factors: 4 × 2 design). Assuming a medium effect size (*f* = 0.25), consistent with prior studies examining multisensory modulation (Wang et al., 2012; Williams LE 2009), a minimum sample size of *N* = 20 was required to achieve 80% power at α = 0.05. Our final sample met this criterion. We selected a medium effect size due to the more exploratory nature of this postural manipulation. Similarly, for Main Experiment 3 (investigating how the modulatory effect of posture on PPI_somatosensory_ varies with different interstimulus intervals), the same design and assumptions yielded a requirement of *N* = 18.

We did not run a power-analysis for Main Experiment 4, but we recruited a number of subjects in line with Main Experiments 2 and 3.

In addition to ANOVA, paired and independent-samples t-tests were employed for specific within- and between-subject comparisons, as appropriate, with assumptions of normality and variance homogeneity assessed prior to testing. Homogeneity of variances was confirmed via Levene’s test for equality of variances. Effect size was calculated by using Cohen’s f^39^. In case of a statistically significant main effect, Bonferroni corrected post-hoc tests were performed.

For linear correlation analysis between PPI_somatosensory_ of the ipsilateral R2 component and posturography data, specifically sway velocity and sway area, data distribution was first evaluated using the Shapiro-Wilk test, which revealed that sway data for the eyes-open condition were not normally distributed (*p* < 0.05). Consequently, a Spearman correlation was used. All statistical analyses were performed in SPSS version 26.

## Supplementary Information

Below is the link to the electronic supplementary material.

Supplementary Material 1

Supplementary Material 2

## Data Availability

All data analysed during this study are included in this published article (and its Supplementary Information files). The datasets generated during the current study are available from the corresponding author on reasonable request.
