# Supplementary material for: Influence of posture on prepulse inhibition and its link to postural control in healthy subjects
Source: Sci Rep. 2025 Dec 20;15:44252. doi: 10.1038/s41598-025-27097-4 (PMC12722243; doi:10.1038/s41598-025-27097-4)
Supplement: Supplementary file 1 — Supplementary Material 1 [file 41598_2025_27097_MOESM1_ESM.docx]

|  |  | | | | | | | | | | | | | |  |  | |
| --- | --- | --- | --- | --- | --- | --- | --- | --- | --- | --- | --- | --- | --- | --- | --- | --- | --- |
|  | |  | | | | | | | **95% Confidence Interval Mean** | | | |  |  |  | | |
|  | | | |  |  | **Mean** | | | **Upper** | | **Lower** | | **Std. Error** | **BR modulation** | **p-value** | | |
| **R1** | | |  | Baseline |  |  | 1.802 |  | 1.610 |  | 0.640 |  | 0.298 | - | - | |  |
|  | | |  | 70 ms |  |  | 2.195 |  | 2.322 |  | 0.766 |  | 0.442 | +14.83 % | P > 0.05 | |  |
|  | | |  | 80 ms |  |  | 2.209 |  | 2.188 |  | 0.686 |  | 0.400 | +23.06 % | P > 0.05 | |  |
|  | | |  | 90 ms |  |  | 2.414 |  | 2.632 |  | 0.826 |  | 0.503 | +30.73 % | P > 0.05 | |  |
|  | | |  | 100 ms |  |  | 2.429 |  | 2.965 |  | 0.819 |  | 0.550 | +24.24 % | P > 0.05 | |  |
|  | | |  | 110 ms |  |  | 2.376 |  | 2.498 |  | 0.859 |  | 0.483 | +25.68 % | P > 0.05 | |  |
|  | | |  | 116 ms |  |  | 1.934 |  | 1.974 |  | 0.624 |  | 0.361 | +6.94 % | P > 0.05 | |  |
|  | | |  | 120 ms |  |  | 2.306 |  | 2.429 |  | 0.787 |  | 0.472 | +23.45 % | P > 0.05 | |  |
|  | | |  |  |  |  |  |  |  |  |  |  |  |  |  | |  |
| **R2** | | |  | Baseline |  |  | 10.156 |  | 1.821 |  | 1.037 |  | 0.387 | - | - | |  |
|  | | |  | 70 ms |  |  | 9.108 |  | 2.828 |  | 1.450 |  | 0.557 | -10.39 % | P > 0.05 | |  |
|  | | |  | 80 ms |  |  | 8.704 |  | 2.330 |  | 1.170 |  | 0.454 | -13.75 % | P > 0.05 | |  |
|  | | |  | 90 ms |  |  | 7.775 |  | 2.566 |  | 1.139 |  | 0.508 | -22.84 % | P = 0.025 | |  |
|  | | |  | 100 ms |  |  | 8.069 |  | 2.472 |  | 1.206 |  | 0.490 | -19.87 % | P > 0.05 | |  |
|  | | |  | 110 ms |  |  | 6.796 |  | 2.450 |  | 1.267 |  | 0.499 | -32.21 % | **P = 0.000123** | |  |
|  | | |  | 116 ms |  |  | 7.536 |  | 3.700 |  | 1.738 |  | 0.726 | -24.78 % | P = 0.009 | |  |
|  | | |  | 120 ms |  |  | 7.926 |  | 2.646 |  | 1.515 |  | 0.549 | -20.68 % | P = 0.07 | |  |
|  | | |  |  |  |  |  |  |  |  |  |  |  |  |  | |  |
| **R2c** | | |  | Baseline |  |  | 9.149 |  | 2.467 |  | 0.916 |  | 0.442 | - | - | |  |
|  | | |  | 70 ms |  |  | 7.367 |  | 2.917 |  | 1.386 |  | 0.562 | - 18.58 % | P > 0.05 | |  |
|  | | |  | 80 ms |  |  | 6.803 |  | 1.983 |  | 1.126 |  | 0.412 | -23.89 % | P > 0.05 | |  |
|  | | |  | 90 ms |  |  | 6.174 |  | 2.238 |  | 1.211 |  | 0.458 | -30.30 % | **P = 0.003** | |  |
|  | | |  | 100 ms |  |  | 6.371 |  | 2.035 |  | 0.633 |  | 0.361 | -28.03% | P = 0.01 | |  |
|  | | |  | 110 ms |  |  | 5.997 |  | 2.312 |  | 1.147 |  | 0.465 | -31.55 % | **P = 0.0023** | |  |
|  | | |  | 116 ms |  |  | 6.469 |  | 2.537 |  | 1.327 |  | 0.517 | -25.95 % | P = 0.026 | |  |
|  | | |  | 120 ms |  |  | 6.104 |  | 1.871 |  | 1.059 |  | 0.389 | -29.88 % | **P = 0.004** | |  |
|  |  | | | | | | | | | | | | |  |  | | |

**Supplementary Table 1: recruitment curve of PPI_somatosensory_ from the leg**. ISI from 70 ms to 120 ms were investigated. Mean, confidence interval, and standard error for amplitude (mV) of R1 and area (mV*ms) of R2 and R2c responses are reported for each condition. Blink Reflex modulation is reported as a percentage, with facilitation represented by positive values and inhibition by negative values. P-values after Bonferroni correction are reported. In bold, ISIs showing a statistically significant inhibition after multiple comparison correction.

|  |  | | | | | | | | | | | | | |  |  | |
| --- | --- | --- | --- | --- | --- | --- | --- | --- | --- | --- | --- | --- | --- | --- | --- | --- | --- |
|  | |  | | | | | | | **95% Confidence Interval Mean** | | | |  |  |  | | |
|  | | | |  |  | **Mean** | | | **Upper** | | **Lower** | | **Std. Error** | **BR modulation** | **p-value** | | |
| **R1** | | |  | Baseline |  |  | 0.910 |  | 1.193 |  | 0.627 |  | 0.131 | - | - | |  |
|  | | |  | 110 ms |  |  | 1.081 |  | 1.379 |  | 0.784 |  | 0.138 | +25.75 % | n.s. | |  |
|  | | |  | 200 ms |  |  | 1.015 |  | 1.278 |  | 0.752 |  | 0.122 | +21.14 % | n.s. | |  |
|  | | |  | 400 ms |  |  | 1.031 |  | 1.264 |  | 0.799 |  | 0.107 | +24.92 % | n.s. | |  |
|  | | |  | 600 ms |  |  | 1.106 |  | 1.373 |  | 0.838 |  | 0.124 | +31.56 % | n.s. | |  |
|  | | |  |  |  |  |  |  |  |  |  |  |  |  |  | |  |
| **R2** | | |  | Baseline |  |  | 10.679 |  | 11.755 |  | 9.602 |  | 0.498 | - | - | |  |
|  | | |  | 110 ms |  |  | 7.768 |  | 9.151 |  | 6.385 |  | 0.640 | -27.58 % | P < 0.005 | |  |
|  | | |  | 200 ms |  |  | 7.847 |  | 8.693 |  | 7.001 |  | 0.392 | -25.96 % | P < 0.005 | |  |
|  | | |  | 400 ms |  |  | 7.669 |  | 8.720 |  | 6.617 |  | 0.487 | -27.75 % | P < 0.005 | |  |
|  | | |  | 600 ms |  |  | 8.263 |  | 9.613 |  | 6.913 |  | 0.625 | -22.73 % | P = 0.022 | |  |
|  | | |  |  |  |  |  |  |  |  |  |  |  |  |  | |  |
| **R2c** | | |  | Baseline |  |  | 9.735 |  | 11.101 |  | 8.369 |  | 0.632 | - | - | |  |
|  | | |  | 110 ms |  |  | 6.924 |  | 8.399 |  | 5.448 |  | 0.683 | - 28.66 % | P = 0.027 | |  |
|  | | |  | 200 ms |  |  | 6.882 |  | 8.043 |  | 5.721 |  | 0.537 | -28.27 % | P = 0.024 | |  |
|  | | |  | 400 ms |  |  | 7.663 |  | 9.117 |  | 6.208 |  | 0.673 | -20.40 % | n.s. | |  |
|  | | |  | 600 ms |  |  | 7.965 |  | 9.371 |  | 6.559 |  | 0.651 | -17.83% | n.s. | |  |
|  |  | | | | | | | | | | | | |  |  | | |

**Supplementary Table 2: recruitment curve of PPI_somatosensory_ from the leg**. ISI 110 ms, 200 ms, 400 ms, and 600 ms were investigated. Mean, confidence interval, and standard error for amplitude (mV) of R1 and area (mV*ms) of R2 and R2c responses are reported for each condition. Blink Reflex modulation is reported as a percentage, with facilitation represented by positive values and inhibition by negative values. P-values after Bonferroni correction are reported.

|  |  | | | |  |  | | | | | | | | | | | | |
| --- | --- | --- | --- | --- | --- | --- | --- | --- | --- | --- | --- | --- | --- | --- | --- | --- | --- | --- |
|  | |  | | | | | | | | | **95% Confidence Interval Mean** | | | |  |  |  | |
|  | | | |  | | |  | **Mean Difference** | | | **Upper** | | **Lower** | | **Std. Error** | **t** | **p-value** | |
| **Supine** | | | | | | |  |  |  |  |  |  |  |  |  |  |  |  |
| R1 | | |  |  | | |  |  | 0.053 |  | 0.158 |  | -0.052 |  | 0.052 | -1.018 | n.s. |  |
| R2 | | |  |  | | |  |  | 0.994 |  | 1.476 |  | 0.5106 |  | 0.240 | -4.146 | P < 0.005 |  |
| R2c | | |  |  | | |  |  | 0.923 |  | 1.608 |  | 0.237 |  | 0.340 | -2.716 | P = 0.009 |  |
|  | | |  |  | | |  |  |  |  |  |  |  |  |  |  |  |  |
| **Hard Surface** | | | | | | |  |  |  |  |  |  |  |  |  |  |  |  |
| R1 | | |  |  | | |  |  | 0.096 |  | 0.212 |  | -0.019 |  | 0.057 | -1.693 | n.s. |  |
| R2 | | |  |  | | |  |  | 1.381 |  | 1.966 |  | 0.796 |  | 0.290 | -4.756 | P < 0.005 |  |
| R2c | | |  |  | | |  |  | 1.579 |  | 2.232 |  | 0.926 |  | 0.324 | -4.872 | P < 0.005 |  |
|  | | |  |  | | |  |  |  |  |  |  |  |  |  |  |  |  |
| **Soft Surface** | | |  |  | | |  |  |  |  |  |  |  |  |  |  |  |  |
| R1 | | |  |  | | |  |  | -0.0822 |  | 0.149 |  | -0.314 |  | 0.115 | 0.713 | n.s. |  |
| R2 | | |  |  | | |  |  | 1.004 |  | 1.631 |  | 0.377 |  | 0.311 | -3.227 | P < 0.005 |  |
| R2c | | |  |  | | |  |  | 1.030 |  | 1.980 |  | 0.085 |  | 0.471 | -0.080 | P = 0.034 |  |
|  | | |  |  | | |  |  |  |  |  |  |  |  |  |  |  |  |
| **Tandem** | | |  |  | | |  |  |  |  |  |  |  |  |  |  |  |  |
| R1 | | |  |  | | |  |  | 0.05 |  | 0.166 |  | -0.066 |  | 0.057 | -0.858 | n.s. |  |
| R2 | | |  |  | | |  |  | 0.733 |  | 1.284 |  | 0.184 |  | 0.273 | -2.688 | P = 0.01 |  |
| R2c | | |  |  | | |  |  | 0.789 |  | 1.36 |  | 0.218 |  | 0.283 | -2.787 | P = 0.008 |  |
|  |  | | | | | | | | | | | | | | |  |  | |

**Supplementary Table 3: difference in latency between unconditioned and conditioned responses.** Mean, confidence interval and standard error for the difference in latency (ms) of R1, R2, and R2c between unconditioned and conditioned responses in each postural condition are reported. T and P-values from paired sampled t-tests are also reported. Both conditioned R2 and R2c latencies are similarly prolonged.

|  |  | | | | | | | | | | | | | | | | | | | | |  | |  | | |  |
| --- | --- | --- | --- | --- | --- | --- | --- | --- | --- | --- | --- | --- | --- | --- | --- | --- | --- | --- | --- | --- | --- | --- | --- | --- | --- | --- | --- |
|  | |  | | | | | | | | **95% Confidence Interval Mean** | | | | | | | |  | |  | |  | | | | |  |
|  | | | |  |  | **Mean** | | | | **Upper** | | | | **Lower** | | | | **Std. Error** | | **F** | | **p-value** | | | | |  |
| **Unconditioned Trials** | | | | |  |  |  |  | |  | |  | |  | |  | |  | |  | |  | | |  | |  |
| **R1** | | |  | Supine |  |  | 10.696 | |  | | 10.959 | |  | | 10.432 | |  | | 0.131 | | F_3,176_ = 0.259 | | 0.855 | | |  | |
|  | | |  | HS |  |  | 10.659 | |  | | 10.909 | |  | | 10.409 | |  | | 0.124 | | - | | - | | |  | |
|  | | |  | SS |  |  | 10.817 | |  | | 11.120 | |  | | 10.515 | |  | | 0.150 | | - | | - | | |  | |
|  | | |  | Tandem |  |  | 10.723 | |  | | 10.975 | |  | | 10.472 | |  | | 0.125 | | - | | - | | |  | |
| **R2** | | |  | Supine |  |  | 31.844 | |  | | 32.766 | |  | | 30.921 | |  | | 0.457 | | F_3,176_ = 0.05 | | 0.985 | | |  | |
|  | | |  | HS |  |  | 31.899 | |  | | 32.626 | |  | | 31.172 | |  | | 0.360 | | - | | - | | |  | |
|  | | |  | SS |  |  | 31.943 | |  | | 32.744 | |  | | 31.142 | |  | | 0.397 | | - | | - | | |  | |
|  | | |  | Tandem |  |  | 31.737 | |  | | 32.463 | |  | | 31.011 | |  | | 0.360 | | - | | - | | |  | |
| **R2c** | | |  | Supine |  |  | 33.786 | |  | | 34.823 | |  | | 32.749 | |  | | 0.514 | | F_3,176_ = 0.185 | | 0.906 | | |  | |
|  | | |  | HS |  |  | 34.090 | |  | | 34.897 | |  | | 33.283 | |  | | 0.400 | | - | | - | | |  | |
|  | | |  | SS |  |  | 34.035 | |  | | 34.975 | |  | | 33.095 | |  | | 0.466 | | - | | - | | |  | |
|  | | |  | Tandem |  |  | 34.267 | |  | | 35.192 | |  | | 33.342 | |  | | 0.459 | | - | | - | | |  | |
|  | | |  |  |  |  |  | |  | |  | |  | |  | |  | |  | |  | |  | | |  | |
| **Conditioned Trials** | | | | |  |  |  | |  | |  | |  | |  | |  | |  | |  | |  | | |  |  |
| **R1** | | |  | Supine |  |  | 10.749 | |  | | 11.010 | |  | | 10.488 | |  | | 0.129 | | F_3,176_ = 0.015 | | 0.997 | | |  |  |
|  | | |  | HS |  |  | 10.756 | |  | | 11.010 | |  | | 10.502 | |  | | 0.125 | | - | | - | | |  |  |
|  | | |  | SS |  |  | 10.735 | |  | | 10.965 | |  | | 10.505 | |  | | 0.114 | | - | | - | | |  |  |
|  | | |  | Tandem |  |  | 10.773 | |  | | 11.035 | |  | | 10.510 | |  | | 0.130 | | - | | - | | |  |  |
| **R2** | | |  | Supine |  |  | 32.837 | |  | | 33.744 | |  | | 31.930 | |  | | 0.450 | | F_3,176_ = 0.646 | | 0.586 | | |  |  |
|  | | |  | HS |  |  | 33.280 | |  | | 34.026 | |  | | 32.533 | |  | | 0.370 | | - | | - | | |  |  |
|  | | |  | SS |  |  | 32.947 | |  | | 33.839 | |  | | 32.055 | |  | | 0.443 | | - | | - | | |  |  |
|  | | |  | Tandem |  |  | 32.470 | |  | | 33.256 | |  | | 31.682 | |  | | 0.391 | | - | | - | | |  |  |
| **R2c** | | |  | Supine |  |  | 34.710 | |  | | 35.842 | |  | | 33.577 | |  | | 0.562 | | F_3,176_ = 0.580 | | 0.629 | | |  |  |
|  | | |  | HS |  |  | 35.669 | |  | | 36.616 | |  | | 34.722 | |  | | 0.470 | | - | | - | | |  |  |
|  | | |  | SS |  |  | 35.065 | |  | | 36.085 | |  | | 34.045 | |  | | 0.506 | | - | | - | | |  |  |
|  | | |  | Tandem |  |  | 35.056 | |  | | 36.168 | |  | | 33.944 | |  | | 0.551 | | - | | - | | |  |  |
|  | | |  |  |  |  |  | |  | |  | |  | |  | |  | |  | |  | |  | | |  |  |
| **Difference vs supine** | | | | | | | | | | | | |  | |  | |  | |  | |  | |  | | |  |  |
| **R1** | | |  | HS |  |  | -0.0435 | |  | | 0.288 | |  | | -0.375 | |  | | 0.10625 | | F_3,176_ = 1.061 | | 0.367 | | |  |  |
|  | | |  | SS |  |  | 0.135 | |  | | 0.4673 | |  | | -0.1966 | |  | | - | | - | | - | | |  |  |
|  | | |  | Tandem |  |  | 0.0033 | |  | | 0.3353 | |  | | -0.2884 | |  | | - | | - | | - | | |  |  |
| **R2** | | |  | HS |  |  | -0.3873 | |  | | 0.8488 | |  | | -1.6235 | |  | | 0.3956 | | F_3,176_ = 0.907 | | 0.439 | | |  |  |
|  | | |  | SS |  |  | -0.0106 | |  | | 1.225 | |  | | -1.2468 | |  | | - | | - | | - | | |  |  |
|  | | |  | Tandem |  |  | 0.2597 | |  | | 1.4959 | |  | | -0.9764 | |  | | - | | - | | - | | |  |  |
| **R2c** | | |  | HS |  |  | -0.6555 | |  | | 0.9422 | |  | | -2.2533 | |  | | 0.5113 | | F_3,176_ = 0.919 | | 0.433 | | |  |  |
|  | | |  | SS |  |  | -0.1073 | |  | | 1.4904 | |  | | -1.7051 | |  | | - | | - | | - | | |  |  |
|  | | |  | Tandem |  |  | 0.1340 | |  | | 1.7317 | |  | | -1.4637 | |  | | - | | - | | - | | |  |  |
|  |  | | | | | | | | | | | | | | | | | | |  | |  | | | | |  |

**Supplementary Table 4: effect of a somatosensory prepulse from the leg on the latency of the blink reflexes responses in different postural conditions.** Mean, confidence interval and standard error for latencies (ms) of R1, R2, and R2c response for unconditioned and conditioned trials are reported for each postural condition. The latency difference for each postural condition relative to the supine position is also reported. Postural changes do not affect the latency of the blink reflexes components.

|  | **n** | **Spearman’s rho** | **P** |  |  |  |  | **n** | **Spearman’s rho** | **P**  **value** | **Z**  **scores** | **P** |
| --- | --- | --- | --- | --- | --- | --- | --- | --- | --- | --- | --- | --- |
|  |  |  |  |  |  |  |  |  |  |  |  |  |
| *Hard Surface – raw values* | | | | | | | *Hard Surface – normalised values* | | | |  |  |
| PPI_somatosensory_ – SV | 45 | 0.250 | 0.01 |  |  |  | PPI_somatosensory_ – SV | 45 | 0.256 | 0.09 | - | - |
| PPI_somatosensory_ – SA | 45 | 0.318 | 0.03 |  |  |  | PPI_somatosensory_ – SA | 45 | 0.321 | 0.032 | - | - |
|  | | | | | | | | | | |  |  |
| *Soft Surface – raw values* | | | | | | | *Soft Surface – normalised values* | | | |  |  |
| PPI_somatosensory_ – SV | 45 | -0.121 | 0.43 |  |  |  | PPI_somatosensory_ – SV | 45 | -0.128 | 0.404 | 1.789 | 0.075 |
| PPI_somatosensory_ – SA | 45 | 0.056 | 0.72 |  |  |  | PPI_somatosensory_ – SA | 45 | 0.055 | 0.719 | 1.273 | 0.204 |
|  | | | | | | | | | | |  |  |
| *Tandem Standing – raw values* | | | | | | | *Tandem Standing – normalised values* | | | |  |  |
| PPI_somatosensory_ – SV | 45 | 0.332 | 0.03 |  |  |  | PPI_somatosensory_ – SV | 45 | 0.344 | 0.021 | 0.167 | 0.868 |
| PPI_somatosensory_ – SA | 45 | 0.280 | 0.06 |  |  |  | PPI_somatosensory_ – SA | 45 | 0.288 | 0.055 | -0.444 | 0.657 |
|  | | | | | | | | | | |  |  |

**Supplementary Table 5: linear correlation between PPI_somatosensory_ and sway parameters**. Spearman's Correlations coefficients and p-values are reported for PPI_somatosensory_ when standing on hard surface, soft surface, and while tandem standing. Z scores for comparison between HS, and SS and TS respectively are reported together with the corresponding p-value.

|  |  | | | | | | | | | | | | | |  |  | | | |
| --- | --- | --- | --- | --- | --- | --- | --- | --- | --- | --- | --- | --- | --- | --- | --- | --- | --- | --- | --- |
|  | |  | | | | | | | **95% Confidence Interval Mean** | | | |  |  |  | | |  |  |
|  | | | |  |  | **Mean** | | | **Upper** | | **Lower** | | **Std. Error** | **F** | **p-value** | | |  |  |
| **Sway Velocity** | | |  | SS vs HS |  |  | 12.132 |  | 15.082 |  | 9.183 |  | 1.464 | F_2,132_ = 43.840 | < 0.001 | |  | |  |
|  | | |  | TS vs HS |  |  | 45.715 |  | 51.546 |  | 39.884 |  | 2.893 | - | - | |  | |  |
|  | | |  | TS vs SS |  |  | 33.583 |  | 39.721 |  | 27.444 |  | 3.046 | - | - | |  | |  |
| **Sway Area** | | |  | SS vs HS |  |  | 6978.422 |  | 10011.047 |  | 3945.796 |  | 1504.750 | F_2,132_ = 8.653 | < 0.001 | |  | |  |
|  | | |  | TS vs HS |  |  | 17398.334 |  | 20878.718 |  | 13917.951 |  | 1726.922 | - | - | |  | |  |
|  | | |  | TS vs SS |  |  | 10419.913 |  | 14707.512 |  | 6132.313 |  | 2127.453 | - | - | |  | |  |
|  |  | | | | | | | | | | | | |  |  | | |  |  |

**Supplementary Table 6: delta changes in sway area and sway velocity between postural tasks.** The table reports mean values, 95% confidence intervals, and standard errors for delta values of sway velocity and sway area. Delta values were calculated by subtracting raw sway area and sway velocity data between conditions: soft surface (SS) minus hard surface (HS), tandem standing (TS) minus HS, and TS minus SS. A repeated measures ANOVA revealed significant differences across these deltas for both sway velocity (post hoc t-tests with Bonferroni correction: SS–HS vs TS–HS:*p* < 0.001; SS–HS vs TS–SS:*p* < 0.001; TS–HS vs TS–SS:*p* = 0.003) and sway area (post hoc t-tests with Bonferroni correction: SS–HS vs TS–HS:*p* < 0.001; SS–HS vs TS–SS:*p* = 0.540; TS–HS vs TS–SS: *p* = 0.021). These results demonstrate a progressive increase in both sway velocity and sway area with increasing task difficulty.

|  | | | |  |  |  |  |  |  |
| --- | --- | --- | --- | --- | --- | --- | --- | --- | --- |
|  | | | |  |  |  |  |  |  |
|  | **F** | **P-value** | | **η_p_2** |  |  | **F** | **P-value** | **η_p_2** |
| **R1 Latency** |  | |  |  |  | **R1 Amplitude** | | |  |
| Eyes | F_1,19_ = 0.664 | 0.427 | | 0.038 |  | Eyes | F_1,19_ = 5.549 | 0.029* | 0.226 |
| Posture | F_2,18_ = 0.304 | 0.742 | | 0.037 |  | Posture | F_2,18_ = 0.143 | 0.867 | 0.016 |
| Eyes*Posture | F_2,18_ = 0.020 | 0.980 | | 0.002 |  | Eyes*Posture | F_2,18_ = 0.373 | 0.694 | 0.040 |
|  |  |  | |  |  |  |  |  |  |
| **R2 Latency** |  | |  |  |  | **R2 Area** | | |  |
| Eyes | F_1,19_ = 0.030 | 0.864 | | 0.002 |  | Eyes | F_1,19_ = 0.033 | 0.858 | 0.002 |
| Posture | F_2,18_ = 0.851 | 0.445 | | 0.096 |  | Posture | F_2,18_ = 13.442 | 0.002* | 0.599 |
| Eyes*Posture | F_2,18_ = 0.305 | 0.741 | | 0.037 |  | Eyes*Posture | F_2,18_ = 1.077 | 0.361 | 0.107 |
|  |  |  | |  |  |  |  |  |  |
| **R2c Latency** |  |  | |  |  | **R2c Area** |  |  |  |
| Eyes | F_1,19_ = 0.049 | 0.827 | | 0.003 |  | Eyes | F_1,19_ = 0.154 | 0.699 | 0.008 |
| Posture | F_2,18_ = 0.785 | 0.473 | | 0.089 |  | Posture | F_2,18_ = 13.147 | 0.003* | 0.594 |
| Eyes*Posture | F_2,18_ = 2.179 | 0.146 | | 0.214 |  | Eyes*Posture | F_2,18_ = 0.032 | 0.969 | 0.003 |
|  | | | |  |  |  |  |  |  |

**Supplementary Table 7: Statistical results of repeated measures ANOVA for all assessed blink reflex parameters**. Significant results are indicated with *.

|  | | | | | | | | | | | | |  |  |
| --- | --- | --- | --- | --- | --- | --- | --- | --- | --- | --- | --- | --- | --- | --- |
|  | | | **Eyes Open** | | | | | **Eyes Closed** | | | | |  |  |
| **Variable** | |  | **Sway Velocity** | | | **Sway Area** | | | **Sway Velocity** | | | **Sway Area** | |  |
| PPI_HS |  | r_s_ |  | 0.398 |  | 0.138 |  | 0.116 | |  | 0.458 | |  | |
|  |  | p-value |  | 0.092 |  | 0.574 |  | 0.635 | |  | 0.049 | |  | |
| PPI_SS |  | r_s_ |  | 0.248 |  | -0.102 |  | 0.123 | |  | -0.039 | |  | |
|  |  | p-value |  | 0.305 |  | 0.677 |  | 0.616 | |  | 0.875 | |  | |
|  |  |  |  |  |  |  |  |  | |  |  | |  | |

**Supplementary Table 8: linear correlation between PPI_somatosensory_ and sway parameters**. Spearman's Correlations coefficients and p-values are reported for PPI_somatosensory_ when standing on hard surface (HS) and soft surface (SS). Each value is then correlated to the corresponding sway parameter (sway velocity and area).

|  | | |  | |  | |  | | |  |
| --- | --- | --- | --- | --- | --- | --- | --- | --- | --- | --- |
|  | | |  | |  | |  | | |  |
|  | **Mean**  **Change**  **(ms)** | **Std. Deviation** | |  |  | **Percentage change (%)** | | **Std. Deviation** | | |
| **R1 Latency** |  | | | | **R1 Amplitude** | | | | | |
| Supine – 80 ms | -0.0864 | 0.4185 | |  | Supine – 80 ms | +41.72 % | | | 33.67 | |
| Supine – 110 ms | 0.0162 | 0.2905 | |  | Supine – 110 ms | +36.18 % | | | 33.39 | |
| HS – 80 ms | -0.0134 | 0.3822 | |  | HS – 80 ms | +19.61 % | | | 33.97 | |
| HS – 110 ms | 0.1116 | 0.3572 | |  | HS – 110 ms | +35.11 % | | | 41.17 | |
| SS – 80 ms | -0.0378 | 0.5155 | |  | SS – 80 ms | +38.59 % | | | 65.67 | |
| SS – 110 ms | +0.1065 | 0.5176 | |  | SS – 110 ms | +42.68 % | | | 68.08 | |
| TS – 80 ms | 0.0918 | 0.4870 | |  | TS – 80 ms | +13.69 % | | | 40.34 | |
| TS – 110 ms | +0.1282 | 0.3692 | |  | TS – 110 ms | +30.59 % | | | 49.83 | |
|  |  |  | |  |  |  | | |  | |
| **R2 Latency** |  | | | | **R2 Area** | | | | | |
| Supine – 80 ms | +0.7817 | 1.6178 | |  | Supine – 80 ms | -10.54 % | | | 21.26 | |
| Supine – 110 ms | +1.2345 | 1.2998 | |  | Supine – 110 ms | -16.10 % | | | 9.7 | |
| HS – 80 ms | +0.8974 | 0.9587 | |  | HS – 80 ms | -22.13 % | | | 12.63 | |
| HS – 110 ms | +1.2760 | 1.6221 | |  | HS – 110 ms | -27.27 % | | | 11.16 | |
| SS – 80 ms | +0.9195 | 1.5880 | |  | SS – 80 ms | -18.12 % | | | 14.98 | |
| SS – 110 ms | +1.2172 | 2.2658 | |  | SS – 110 ms | -18.52 % | | | 14.41 | |
| TS – 80 ms | +0.6721 | 1.4709 | |  | TS – 80 ms | -19.35 % | | | 26.11 | |
| TS – 110 ms | +1.0387 | 1.9831 | |  | TS – 110 ms | -13.58 % | | | 12.93 | |
|  |  |  | |  |  |  | | |  | |
| **R2c Latency** |  | | | | **R2c Area** |  | | |  | |
| Supine – 80 ms | +1.3663 | 1.7426 | |  | Supine – 80 ms | -6.39 % | | | 25.04 | |
| Supine – 110 ms | +1.3885 | 1.5047 | |  | Supine – 110 ms | -19.42 % | | | 7.47 | |
| HS – 80 ms | +0.8181 | 1.1737 | |  | HS – 80 ms | -18.96 % | | | 19.30 | |
| HS – 110 ms | +1.3461 | 1.6363 | |  | HS – 110 ms | -29.33 % | | | 10.21 | |
| SS – 80 ms | +0.6624 | 1.9891 | |  | SS – 80 ms | -19.17 % | | | 21.87 | |
| SS – 110 ms | +1.2526 | 3.7025 | |  | SS – 110 ms | -16.47 % | | | 9.08 | |
| TS – 80 ms | +0.2530 | 1.1430 | |  | TS – 80 ms | -17.19 % | | | 34.55 | |
| TS – 110 ms | +1.2621 | 2.1822 | |  | TS – 110 ms | -13.19 % | | | 12.05 | |
|  | | |  | |  | |  | | |  |

**Supplementary Table 9: postural modulation of PPI_somatosensory_ according to the ISIs deployed for each blink reflex response**. Mean changes and standard deviation in latency (ms) for R1, R2, and R2c are reported according to the specified ISI (80 ms or 110 ms) and according to the postural condition. Similarly, relative changes (%) and standard deviation for the R1 amplitude (mV) and R2 and R2c area (mV*ms) are reported. HS: hard surface; SS: soft surface, TS: tandem standing.

|  | | |  | |  | |  | | |  |  |  |
| --- | --- | --- | --- | --- | --- | --- | --- | --- | --- | --- | --- | --- |
|  | | |  | |  | |  | | |  |  |  |
|  | **Mean**  **Change**  **(ms)** | **Std. Deviation** | |  |  | **Percentage change (%)** | | **Std. Deviation** | | | **t** | **p-value** |
| **R1 Latency** |  | | | | **R1 Amplitude** | | | | | |  |  |
| Supine – 110 ms | +0.0763 | 0.3250 | |  | Supine – 110 ms | +39.75 % | | | 63.22 | | 0.622 | n.s |
| Supine – 200 ms | +0.0611 | 0.4220 | |  | Supine – 200 ms | +31.86 % | | | 56.18 | | - | - |
| HS – 110 ms | +0.1395 | 0.3127 | |  | HS – 110 ms | +32.25 % | | | 73.10 | | 0.141 | n.s |
| HS – 200 ms | +0.1500 | 0.3269 | |  | HS – 200 ms | +30.41 % | | | 42.74 | | - | - |
| SS – 110 ms | +0.0116 | 0.4457 | |  | SS – 110 ms | +18.15 % | | | 38.52 | | -1.712 | n.s |
| SS – 200 ms | +0.0516 | 0.2459 | |  | SS – 200 ms | +36.74 % | | | 57.91 | | - | - |
| TS – 110 ms | -0.0174 | 0.3943 | |  | TS – 110 ms | +36.06 % | | | 28.97 | | -0.844 | n.s |
| TS – 200 ms | +0.0832 | 0.2777 | |  | TS – 200 ms | +47.50 % | | | 57.59 | | - | - |
|  |  |  | |  |  |  | | |  | |  |  |
| **R2 Latency** |  | | | | **R2 Area** | | | | | |  |  |
| Supine – 110 ms | +0.5016 | 1.9174 | |  | Supine – 110 ms | -19.71 % | | | 16.00 | | 2.802 | P = 0.012 |
| Supine – 200 ms | +0.7284 | 2.1925 | |  | Supine – 200 ms | -37.06 % | | | 28.67 | | - | - |
| HS – 110 ms | +0.5053 | 1.65365 | |  | HS – 110 ms | -35.79 % | | | 20.15 | | 1.654 | P = 0.116 |
| HS – 200 ms | +0.6668 | 1.8377 | |  | HS – 200 ms | -43.37 % | | | 23.86 | | - | - |
| SS – 110 ms | +0.2132 | 1.1911 | |  | SS – 110 ms | -18.01 % | | | 22.11 | | 4.782 | P < 0.001 |
| SS – 200 ms | +1.1105 | 1.3827 | |  | SS – 200 ms | -37.61 % | | | 19.32 | | - | - |
| TS – 110 ms | +0.4747 | 1.5657 | |  | TS – 110 ms | -22.31 % | | | 18.60 | | 1.968 | P = 0.065 |
| TS – 200 ms | +1.5863 | 1.5045 | |  | TS – 200 ms | -31.16 % | | | 28.94 | | - | - |
|  |  |  | |  |  |  | | |  | |  |  |
| **R2c Latency** |  | | | | **R2c Area** |  | | |  | |  |  |
| Supine – 110 ms | +0.5768 | 1.9269 | |  | Supine – 110 ms | -26.59 % | | | 26.62 | | 2.871 | P = 0.010 |
| Supine – 200 ms | +0.7037 | 1.9097 | |  | Supine – 200 ms | -41.06 % | | | 33.11 | | - | - |
| HS – 110 ms | +0.3916 | 1.3853 | |  | HS – 110 ms | -27.75 % | | | 25.66 | | 3.660 | P = 0.002 |
| HS – 200 ms | +0.1879 | 1.6685 | |  | HS – 200 ms | -39.59 % | | | 24.76 | | - | - |
| SS – 110 ms | -0.1447 | 1.6146 | |  | SS – 110 ms | -23.89 % | | | 26.66 | | 4.089 | P = 0.001 |
| SS – 200 ms | +0.5874 | 1.9102 | |  | SS – 200 ms | -39.53 % | | | 24.45 | | - | - |
| TS – 110 ms | +0.2832 | 1.5053 | |  | TS – 110 ms | -30.63 % | | | 23.80 | | 2.050 | P = 0.055 |
| TS – 200 ms | +1.5005 | 1.8519 | |  | TS – 200 ms | -39.12 % | | | 23.57 | | - | - |
|  | | |  | |  | |  | | |  |  |  |

**Supplementary Table 10: postural modulation of PPI_somatosensory_ according to the ISIs deployed for each blink reflex response**. Mean changes and standard deviation in latency (ms) for R1, R2, and R2c are reported according to the specified ISI (110 ms or 200 ms) and according to the postural condition. Similarly, relative changes (%) and standard deviation for the R1 amplitude (mV) and R2 and R2c area (mV*ms) are reported. HS: hard surface; SS: soft surface, TS: tandem standing.

|  |  | | | |  |  | | | | | | | | | | | | |
| --- | --- | --- | --- | --- | --- | --- | --- | --- | --- | --- | --- | --- | --- | --- | --- | --- | --- | --- |
|  | |  | | | | | | | | | **95% Confidence Interval Mean** | | | |  |  |  | |
|  | | | |  | | |  | **Mean Difference** | | | **Upper** | | **Lower** | | **Std. Error** | **t** | **p-value** | |
| **Supine** | | | | | | |  |  |  |  |  |  |  |  |  |  |  |  |
| R1 | | |  |  | | |  |  | 0.2050 |  | 0.4523 |  | -0.0423 |  | 0.1181 | 1.735 | n.s. |  |
| R2 | | |  |  | | |  |  | -2.2880 |  | -1.3209 |  | -3.2550 |  | 0.4620 | -4.952 | P < 0.001 |  |
| R2c | | |  |  | | |  |  | -1.8715 |  | -0.3713 |  | -3.3716 |  | 0.7167 | -2.611 | P = 0.017 |  |
|  | | |  |  | | |  |  |  |  |  |  |  |  |  |  |  |  |
| **Hard Surface** | | | | | | |  |  |  |  |  |  |  |  |  |  |  |  |
| R1 | | |  |  | | |  |  | 0.0955 |  | 0.2751 |  | -0.0841 |  | 0.0858 | 1.113 | n.s. |  |
| R2 | | |  |  | | |  |  | -1.3920 |  | -0.2989 |  | -2.4851 |  | 0.5222 | -2.665 | P = 0.015 |  |
| R2c | | |  |  | | |  |  | -0.2465 |  | 0.8163 |  | -1.3093 |  | 0.5078 | -0.485 | n.s. |  |
|  | | |  |  | | |  |  |  |  |  |  |  |  |  |  |  |  |
| **Soft Surface** | | |  |  | | |  |  |  |  |  |  |  |  |  |  |  |  |
| R1 | | |  |  | | |  |  | 0.0395 |  | 0.2424 |  | -0.1634 |  | 0.0969 | 0.407 | n.s. |  |
| R2 | | |  |  | | |  |  | -1.7890 |  | -1.0365 |  | -2.5414 |  | 0.3595 | -4.976 | P < 0.001 |  |
| R2c | | |  |  | | |  |  | -1.6765 |  | -1.0008 |  | -2.3521 |  | 0.3228 | -5.193 | P < 0.001 |  |
|  | | |  |  | | |  |  |  |  |  |  |  |  |  |  |  |  |
| **Tandem** | | |  |  | | |  |  |  |  |  |  |  |  |  |  |  |  |
| R1 | | |  |  | | |  |  | -0.1240 |  | 0.1265 |  | -0.3745 |  | 0.1197 | -1.036 | n.s. |  |
| R2 | | |  |  | | |  |  | -1.2505 |  | -0.3168 |  | -2.1841 |  | 0.4460 | -2.803 | P = 0.011 |  |
| R2c | | |  |  | | |  |  | -0.6045 |  | 0.1086 |  | -1.3176 |  | 0.3407 | -1.774 | n.s. |  |
|  |  | | | | | | | | | | | | | | |  |  | |

**Supplementary Table 11: difference in latency between unconditioned and conditioned responses during the PPI_auditory_ task.** Mean, confidence interval and standard error for the difference in latency (ms) of R1, R2, and R2c between unconditioned and conditioned responses in each postural condition are reported. T and P-values from paired sampled t-tests are also reported. Both conditioned R2 and R2c latencies are similarly prolonged.

|  |  | | | | | | | | | | | | | | | | | |  | |  | |
| --- | --- | --- | --- | --- | --- | --- | --- | --- | --- | --- | --- | --- | --- | --- | --- | --- | --- | --- | --- | --- | --- | --- |
|  | |  | | | | | | | **98.75% Confidence Interval Mean** | | | | | |  | |  | |  | | | |
|  | | | |  |  | **Mean** | | | **Upper** | | | **Lower** | | | **Std. Error** | | **F** | | **p-value** | | | |
| **Difference vs supine** | | | | | | | | | | |  | |  |  | |  | |  | |  | |  |
| **R1** | | |  | HS |  |  | -0.1090 |  | | 0.3695 |  | | -0.5875 |  | | 0.1501 | | F_3,79_ = 1.665 | | n.s. | |  |
|  | | |  | SS |  |  | -0.1650 |  | | 0.3135 |  | | -0.6435 |  | | - | | - | | - | |  |
|  | | |  | Tandem |  |  | -0.3285 |  | | 0.1500 |  | | -0.8070 |  | | - | | - | | - | |  |
| **R2** | | |  | HS |  |  | 0.8960 |  | | 2.9301 |  | | -1.1381 |  | | 0.6381 | | F_3,79_ = 1.062 | | n.s. | |  |
|  | | |  | SS |  |  | 0.4990 |  | | 2.5331 |  | | -1.5351 |  | | - | | - | | - | |  |
|  | | |  | Tandem |  |  | 1.0375 |  | | 3.0716 |  | | -0.9966 |  | | - | | - | | - | |  |
| **R2c** | | |  | HS |  |  | 1.6245 |  | | 3.8693 |  | | -0.6203 |  | | 0.7042 | | F_3,79_ = 2.555 | | n.s. | |  |
|  | | |  | SS |  |  | 0.1940 |  | | 2.4388 |  | | -2.0508 |  | | - | | - | | - | |  |
|  | | |  | Tandem |  |  | 1.2660 |  | | 3.5108 |  | | -0.9788 |  | | - | | - | | - | |  |
|  |  | | | | | | | | | | | | | | | |  | |  | | | |

**Supplementary Table 12: effect of an auditory prepulse on the latency of the blink reflexes responses in different postural conditions.** Mean, confidence interval and standard error for the latency difference (ms) for each postural condition relative to the supine position are reported. Postural changes do not affect the latency of any blink reflexes components.

|  |  | | | |  |  | | | | | | | | | | | | |
| --- | --- | --- | --- | --- | --- | --- | --- | --- | --- | --- | --- | --- | --- | --- | --- | --- | --- | --- |
|  | |  | | | | | | | | | **95% Confidence Interval Mean** | | | |  |  |  | |
|  | | | |  | | |  | **Mean**  **Percentage Change** | | | **Upper** | | **Lower** | | **Std. Error** | **F** | **p-value** | |
| **R1 Amplitude** | | | | | | |  |  |  |  |  |  |  |  |  | 0.929 | n.s. |  |
| Supine | | |  |  | | |  |  | +38.69 % |  | +68.06 % |  | +09.32 % |  | 0.140 | - | - |  |
| HS-EO | | |  |  | | |  |  | +39.95 % |  | +52.04 % |  | +27.85 % |  | 0.058 | - | - |  |
| SS-EO | | |  |  | | |  |  | +56.00 % |  | +92.51 % |  | +19.49 % |  | 0.174 | - | - |  |
| TS-EO | | |  |  | | |  |  | +27.87 % |  | +41.77 % |  | +13.97 % |  | 0.066 | - | - |  |
|  | | |  |  | | |  |  |  |  |  |  |  |  |  |  |  |  |
| **R2 Area** | | | | | | |  |  |  |  |  |  |  |  |  | 4.476 | P = 0.006 |  |
| Supine | | |  |  | | |  |  | -22.70 % |  | -16.35 % |  | -29.05 % |  | 0.030 | - | - |  |
| HS-EO | | |  |  | | |  |  | -32.85 % |  | -26.84 % |  | -38.86 % |  | 0.028 | - | - |  |
| SS-EO | | |  |  | | |  |  | -17.80 % |  | -09.08 % |  | -26.52 % |  | 0.042 | - | - |  |
| TS-EO | | |  |  | | |  |  | -14.50 % |  | -04.59 % |  | -24.41 % |  | 0.047 | - | - |  |
|  | | |  |  | | |  |  |  |  |  |  |  |  |  |  |  |  |
| **R2c Area** | | |  |  | | |  |  |  |  |  |  |  |  |  | 5.266 | p = 0.002 |  |
| Supine | | |  |  | | |  |  | -20.69 % |  | -14.89 % |  | -26.49 % |  | 0.027 | - | - |  |
| HS-EO | | |  |  | | |  |  | -28.53 % |  | -21.88 % |  | -35.18 % |  | 0.031 | - | - |  |
| SS-EO | | |  |  | | |  |  | -07.93 % |  | +01.33 % |  | -17.19 % |  | 0.044 | - | - |  |
| TS-EO | | |  |  | | |  |  | -10.91 % |  | +00.56 % |  | -22.37 % |  | 0.054 | - | - |  |
|  |  | | | | | | | | | | | | | | |  |  | |

**Supplementary Table 13: postural modulation of the magnitude of PPI_auditory_ for each blink reflex response.** Mean of percentage change, confidence interval, and standard error of the amount of inhibition for each blink reflex component is reported according to posture. Positive values reflect a facilitation, whereas negative values reflect an inhibition of the blink reflex responses. R1 amplitude shows a tendency towards facilitation similar in all the different postural conditions. Conversely, R2 and R2c area are modulated according to posture, with a stronger inhibition in the HS-EO condition and a weaker inhibition in the SS and TS-EO condition.

|  | | | | | | | |
| --- | --- | --- | --- | --- | --- | --- | --- |
|  | | | | | | | |
| **Variable** | |  | **Sway Velocity** | | | **Sway Area** | |
| PPI_HS |  | r_s_ |  | -0.087 |  | 0.057 |  |
|  |  | p-value |  | 0.716 |  | 0.811 |  |
| PPI_SS |  | r_s_ |  | -0.063 |  | 0.049 |  |
|  |  | p-value |  | 0.794 |  | 0.838 |  |
| PPI_TS |  | r_s_ |  | 0.411 |  | 0.522 |  |
|  |  | p-value |  | 0.072 |  | **0.018** |  |
|  |  |  |  |  |  |  |  |
|  | | | | | | | |

**Supplementary Table 14: linear correlation between PPI_auditory_ and sway parameters**. Spearman's Correlations coefficients and p-values are reported for aPPI when standing on hard surface (HS), soft surface (SS), and tandem standing (TS).

|  | | | | | | | | | | | |
| --- | --- | --- | --- | --- | --- | --- | --- | --- | --- | --- | --- |
|  | | | | | | | | 95% Confidence Interval Mean | | | |
|  | |  | | Mean | | Std. Error of Mean | | Upper | | Lower | |
| R1_PPI_auditory_ |  | Supine |  | 1.410 |  | 0.146 |  | 1.696 |  | 1.123 |  |
|  |  | HS |  | 1.419 |  | 0.057 |  | 1.531 |  | 1.306 |  |
|  |  | SS |  | 1.585 |  | 0.182 |  | 1.942 |  | 1.228 |  |
|  |  | TS |  | 1.274 |  | 0.070 |  | 1.411 |  | 1.137 |  |
| R1_ PPI_somatosensory_ |  | Supine |  | 1.362 |  | 0.077 |  | 1.512 |  | 1.212 |  |
|  |  | HS |  | 1.351 |  | 0.094 |  | 1.536 |  | 1.166 |  |
|  |  | SS |  | 1.427 |  | 0.156 |  | 1.733 |  | 1.121 |  |
|  |  | TS |  | 1.306 |  | 0.114 |  | 1.530 |  | 1.082 |  |
| R2_ PPI_auditory_ |  | Supine |  | 0.776 |  | 0.032 |  | 0.839 |  | 0.713 |  |
|  |  | HS |  | 0.689 |  | 0.025 |  | 0.739 |  | 0.639 |  |
|  |  | SS |  | 0.815 |  | 0.043 |  | 0.900 |  | 0.730 |  |
|  |  | TS |  | 0.861 |  | 0.050 |  | 0.958 |  | 0.764 |  |
| R2_ PPI_somatosensory_ |  | Supine |  | 0.849 |  | 0.020 |  | 0.888 |  | 0.810 |  |
|  |  | HS |  | 0.713 |  | 0.032 |  | 0.776 |  | 0.651 |  |
|  |  | SS |  | 0.802 |  | 0.031 |  | 0.862 |  | 0.741 |  |
|  |  | TS |  | 0.882 |  | 0.020 |  | 0.922 |  | 0.842 |  |
| R2c_ PPI_auditory_ |  | Supine |  | 0.795 |  | 0.029 |  | 0.852 |  | 0.738 |  |
|  |  | HS |  | 0.733 |  | 0.027 |  | 0.787 |  | 0.680 |  |
|  |  | SS |  | 0.919 |  | 0.047 |  | 1.010 |  | 0.828 |  |
|  |  | TS |  | 0.905 |  | 0.056 |  | 1.014 |  | 0.796 |  |
| R2c PPI_somatosensory_ |  | Supine |  | 0.765 |  | 0.027 |  | 0.817 |  | 0.713 |  |
|  |  | HS |  | 0.694 |  | 0.028 |  | 0.750 |  | 0.639 |  |
|  |  | SS |  | 0.825 |  | 0.024 |  | 0.872 |  | 0.778 |  |
|  |  | TS |  | 0.851 |  | 0.033 |  | 0.916 |  | 0.787 |  |
|  | | | | | | | | | | | |

**Supplementary Table 15: comparison of auditory and somatosensory PPI according to posture**. Mean changes and standard error for the R1 amplitude (mV) and R2 and R2c area (mV*ms) are reported for both PPI_auditory and_ PPI_somatosensory_. HS: hard surface; SS: soft surface, TS: tandem standing.
