# Supplementary material for: Influence of posture on prepulse inhibition and its link to postural control in healthy subjects
Source: Sci Rep. 2025 Dec 20;15:44252. doi: 10.1038/s41598-025-27097-4 (PMC12722243; doi:10.1038/s41598-025-27097-4)
Supplement: Supplementary file 2 — Supplementary Material 2 [file 41598_2025_27097_MOESM2_ESM.docx]

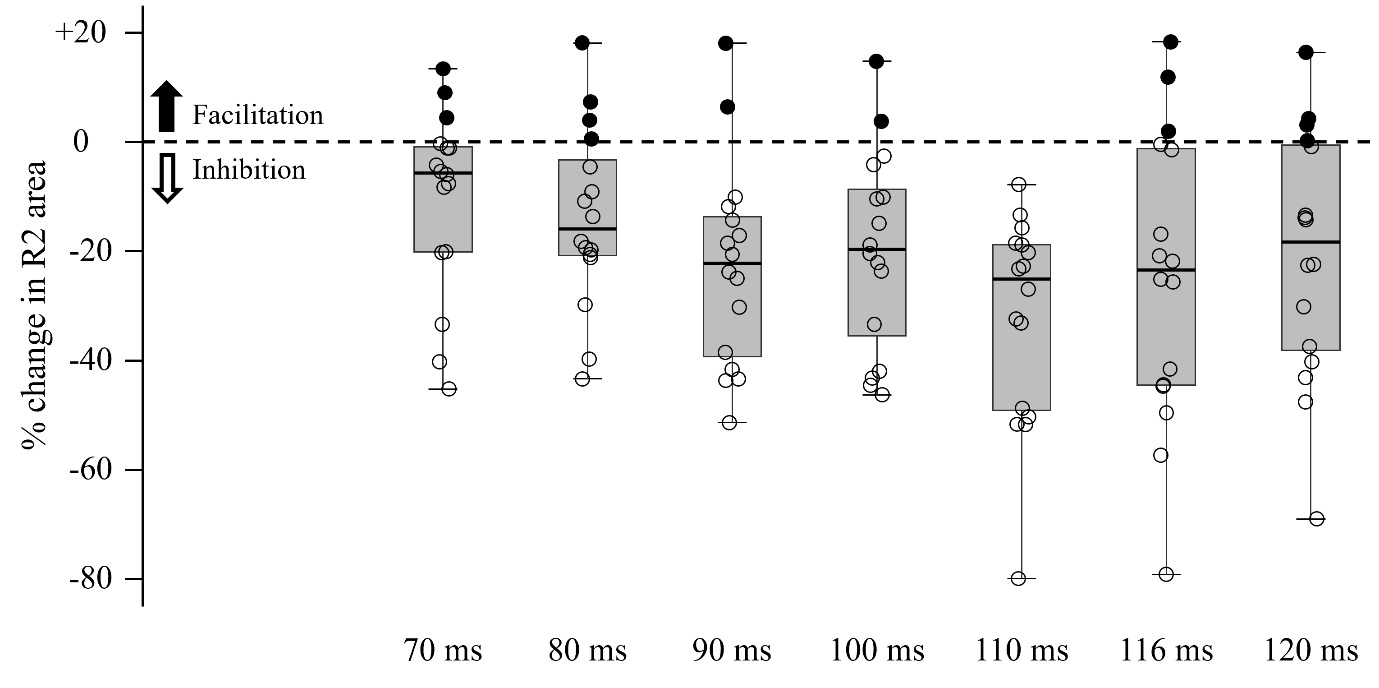


**Supplementary Figure 1: modulation of R2 area according to the applied interstimulus interval**. Relative changes (%) in the R2 area are illustrated. Single dots represent the amount of facilitation (black filled circles) or inhibition (empty circles) for each participant at each specific interstimulus interval. At an interstimulus interval of 110 ms, all participants consistently exhibit an inhibition in the R2 area of the conditioned response.


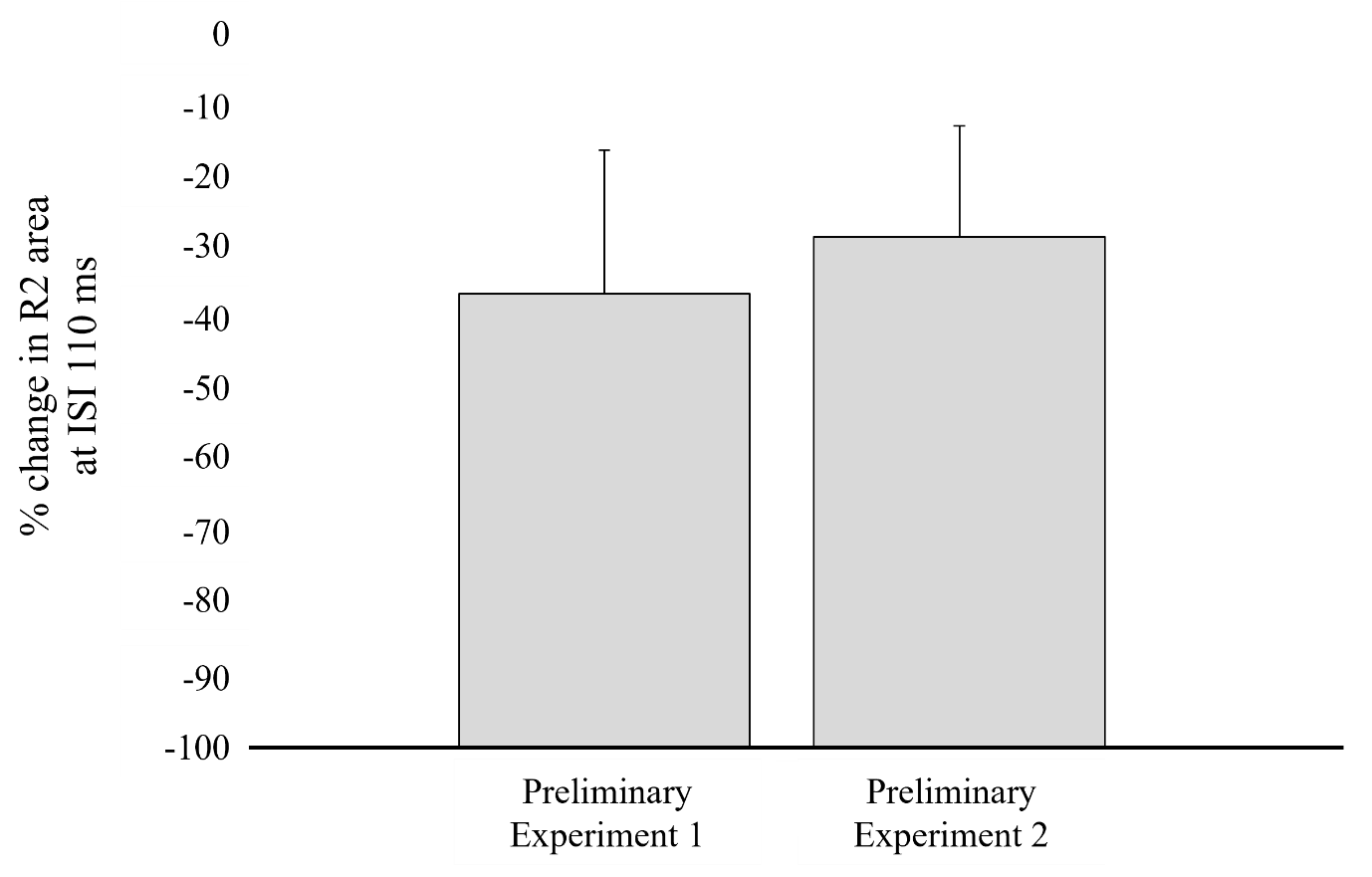


**Supplementary Figure 2: reliability of sPPI of R2 area at 110 ms**. Relative changes (%) in the R2 are illustrated. At an interstimulus interval of 110 ms, no statistically significant differences were noted between groups. PPI_somatosensory_ at 110 ms was 36.33 % and 28.38 % during preliminary experiment 1 and 2 respectively.


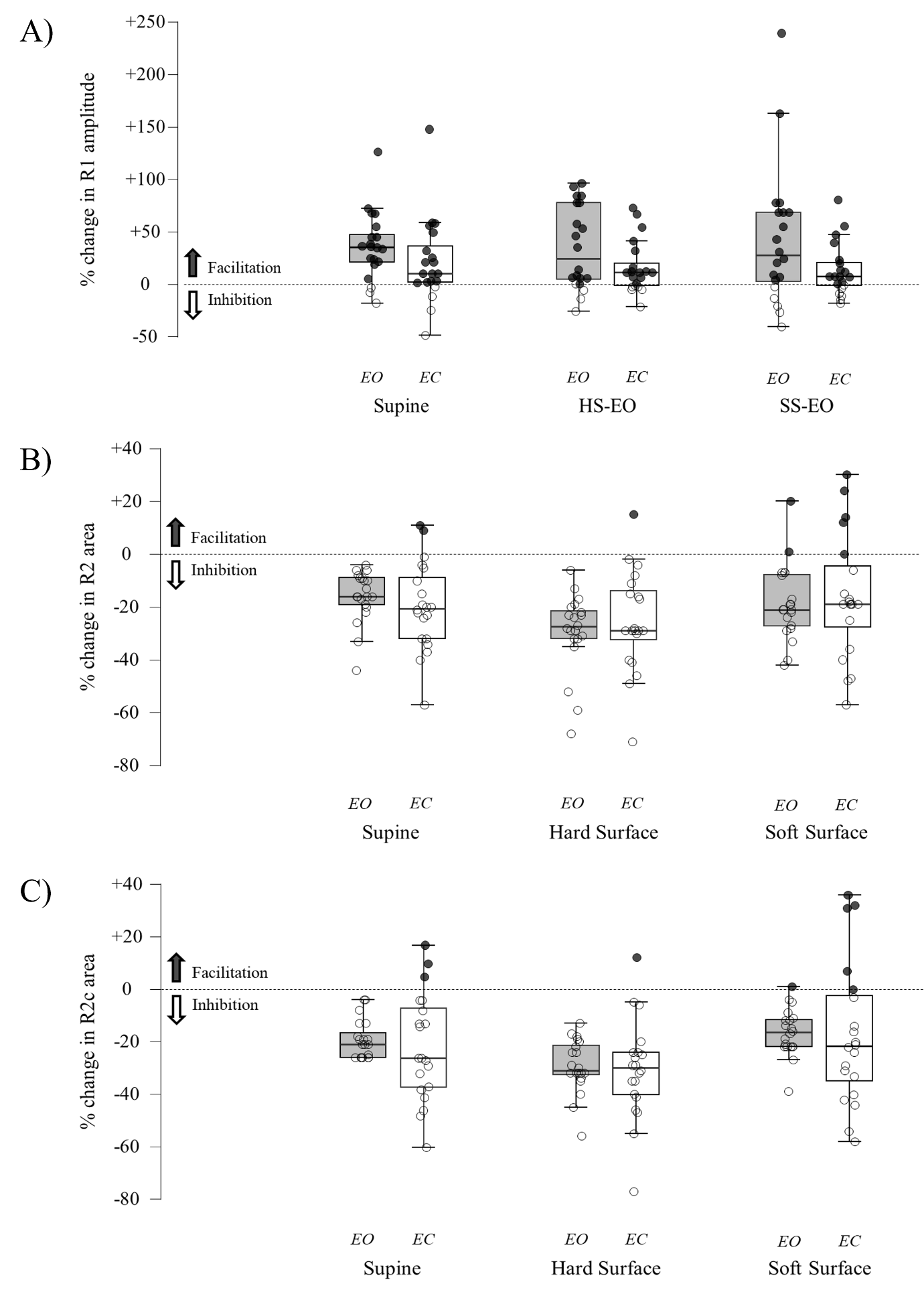


**Supplementary Figure 3: modulation of the R2 and R2c area according to posture and visual feedback.** Relative changes (%) in the R1 amplitude (mV) (A), R2 (B) and R2c (C) area (mV*ms) are illustrated. A statistically significant difference after Bonferroni correction is present between supine and hard surface and hard-surface and soft-surface regardless the visual feedback. EO: eyes open; EC: eyes closed. *: p< 0.0125.


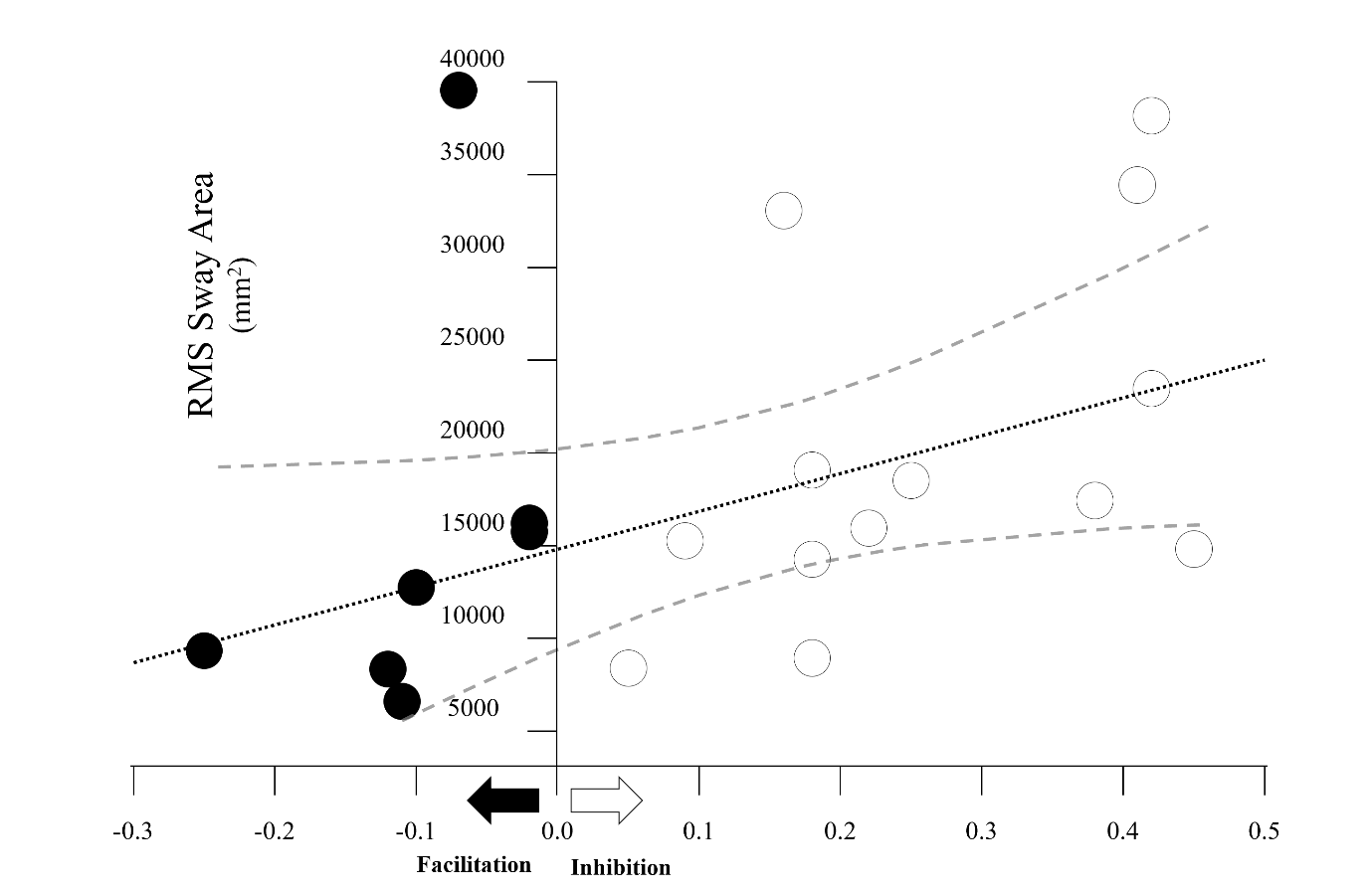


**Supplementary Figure 4: linear correlation between PPI_auditory_ and sway area.** There is a positive linear correlation between PPI_auditory_ and sway area while tandem standing. A trend towards a positive correlation is also present between PPI_auditory_ and sway velocity (not reported). The blacked dotted line represents the regression line, while the two blue dashed lines represent the 95% confident interval.
